# Deep magma underpressure and connectivity drive large dike intrusions

**DOI:** 10.1126/sciadv.aed6073

**Published:** 2026-07-08

**Authors:** Carolina Pagli, Alessandro La Rosa, Simone Cesca, Torsten Dahm, Hua Wang, Manuela Bonano, Pasquale Striano, Pinar Büyükakpınar, Eleonora Rivalta, Derek Keir, Francesco Casu, Atalay Ayele, Elias Lewi

**Affiliations:** ^1^ Department of Earth Sciences, University of Pisa, Pisa, Italy.; ^2^ GFZ Helmholtz Centre for Geosciences, Potsdam, Germany.; ^3^ University of Potsdam, Potsdam, Germany.; ^4^ College of Natural Resources and Environment, South China Agricultural University, Guangzhou, China.; ^5^ Institute for Electromagnetic Sensing of Environment (IREA), National Research Council (CNR), Naples-Milan, Italy.; ^6^ Department of Physics and Astronomy, University of Bologna, Bologna, Italy.; ^7^ School of Ocean and Earth Science, University of Southampton, Southampton, UK.; ^8^ Department of Earth Sciences, University of Florence, Florence, Italy.; ^9^ Institute of Geophysics, Space Science and Astronomy (IGSSA), Addis Ababa University, Addis Ababa, Ethiopia.

## Abstract

Large dikes are the main mechanism of crustal extension in volcanic areas, but the processes in the underlying magma system that supply the required volumes remain unclear. We show that 1.4 cubic kilometers of magma propagated under the Ethiopian rift in December 2024 and continued for ~3 months. Geodesy and seismicity reveal that the dike was fed from a network of magma reservoirs between 6 to 12 kilometers in depth with pathways rapidly forming between them. We calculate pressure changes in the reservoirs and show that underpressure developed in the deeper portion, creating the conditions to drain large magma volumes. We find that tectonic stress and availability of magma alone are not enough to drive intrusion of massive dikes. These events will start only after magma connectivity and deep underpressure develop. Similar conditions may be important for the transfer of large magma volumes from the mantle and the formation of large igneous provinces.

## Teaser

Intrusion of massive dike shows that deep magma underpressure and connectivity drive large magma transfer.

## INTRODUCTION

The existence of large dikes with volumes of km^3^ on Earth and their importance in creating new crust along rifts and ridges is well established (*1*–*5*). However, the formation of these dikes has been rarely observed with modern geophysical methods (*3*–*7*), and the conditions in the magma domain required to supply large dike volumes have been difficult to resolve. Conceptual models of magmatic systems suggest that they consist of a mix of crystals and melt throughout the crust (*8*). The challenge has been to resolve how vertically extensive the melt storage region is, how reservoirs interact, and over what timescale melt lenses connect (*9*–*11*). It therefore remains unclear what are the magma storage conditions that allow melt transport feeding large propagating dikes. Here, we present the 2024–2025 Fentale-Dofen dike intrusion in the Main Ethiopian Rift (MER), an event that lasted about 3 months from December 2024 to March 2025 during which 1.4 km^3^ of magma moved from deep to shallow crustal levels. The intrusion was imaged with Interferometric Synthetic Aperture Radar (InSAR) and seismicity at an exceptional temporal resolution allowing us to provide clear evidence of the full range of magma transport processes.

**Fig. 1.**
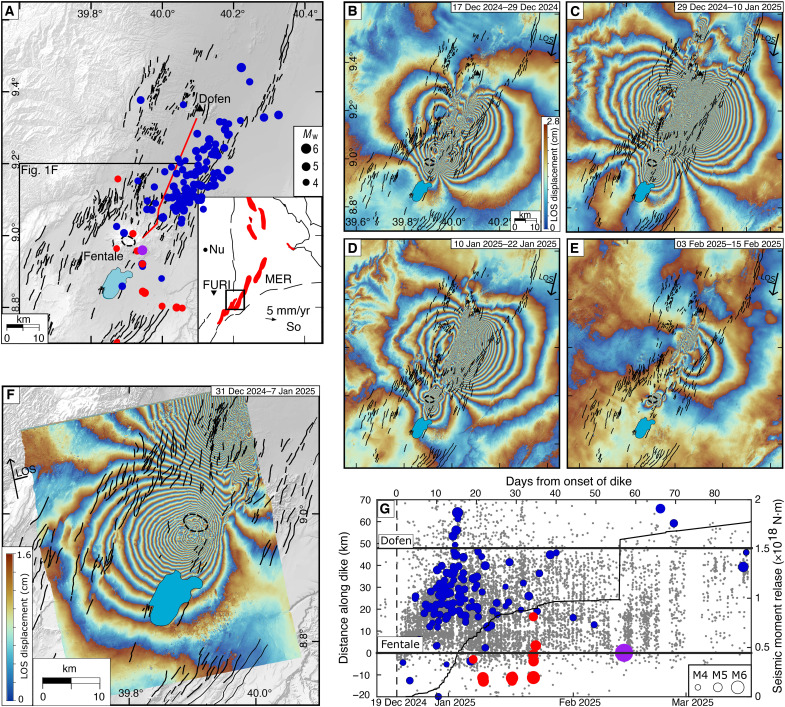
InSAR and seismicity in the FD segment, MER. (**A**) Shaded relief of FD. The dashed line is the Fentale caldera, the red line is the dike, black lines are faults, the blue outline is lake Beseka. Blue dots are the dike-induced earthquakes, red dots are the sill related earthquakes, and the *M*_w_ 5.8 earthquake under Fentale is plotted in purple. The inset shows the location of the dike area (black rectangle), the seismic station FURI (black inverted triangle), the magmatic segments (red ovals), the MER and the extension of Somalia plate (So) with respect to Nubia (Nu) in mm per year (mm/yr). (**B** to **E**) Wrapped S1 interferograms, covering the same area as in (A) and (**F**) wrapped CSK interferogram. The time period spanned by the interferograms is in the top right corner. The values in all the interferograms are in the satellite line-of-sight (LOS) direction, and positive values mean a range increase (motion away from the satellite), such as caused by deflation. The satellite orbit and the LOS are shown by the black arrow with the tick. (**G**) Seismicity along the dike (dots); blue dots are the dike-induced earthquakes, and red dots are the Fentale sill related earthquakes as in (A). The location of Fentale and Dofen along the dike is marked, the black line is the cumulative seismic moment release.

The MER formed during the past ~20 million years (Ma) from ~E-W extension between the Nubian and Somalian plates at ~5 mm/year (*12*, *13*). Since ~2 Ma, faulting and volcanism have localized to a series of NNE-striking axial volcanic segments (Fig. 1A) (*14*, *15*). The Fentale-Dofen segment (FD) consists of a ~60-km-long graben with fissural flows, faults and two volcanoes, the Fentale caldera at the southern tip of the segment, and the Dofen rifted shield volcano at the northern end (Fig. 1A) (*15*–*17*). The crust beneath the FD has a thickness of 25 to 30 km (*18*). According to historical records, the most recent eruption occurred in 1770 to 1808 as a basaltic lava flow from a 5-km fissure cutting Sabober cone, south of Fentale (*15*). More recently in 2015, a ~10-km-long NE-directed rhyolite dike intruded over ~6 months near Fentale volcano (*19*, *20*). A period of repeated dike intrusions, called a rifting episode (*1*), started in FD in September 2024, when a ~15-km-long dike opened by ~2 m between September and November (*21*). The intrusion was preceded by a 3-year inflation at Fentale volcano, but no deflation during diking was observed (*21*). Activity resumed on 19 December 2024 when global earthquake catalogs showed increased seismicity near Fentale volcano. Within a few days, earthquakes started migrating northward and by 3 January had reached Dofen volcano, ~45 km to the north. Intense seismicity up to magnitude 5.8 continued along the whole length of the volcanic segment and ~20 km beyond it. Then, the rate of earthquakes decreased, stopping in early March 2025.

We analyzed the ground deformation with InSAR, using C band and X band images acquired by the Sentinel-1 (S1) and the COSMO-SkyMed (CSK) satellites, respectively (Fig. 1, B to F). The S1 and CSK dataset complement each other in spatiotemporal resolution and imaging geometries. S1 covers relatively large areas (~250 km in width) with limited temporal resolution (revisit time of 12 days), while CSK covers small areas (~40 km in width) at high temporal resolution (revisit time as short as 1 day). Interferograms and maps of range offsets (ROs) were used for modeling the spatial and temporal evolution of the deformation. The modeling was carried out assuming sources of deformation within a uniform elastic half space. We complemented the InSAR results with analysis of global seismic data. First, we compiled Global Centroid Moment Tensor (CMT), National Earthquake Information Center (NEIC), Geofon, and International Seismological Centre (ISC) catalogs and found 285 earthquakes near the FD segment during the intrusion. Then, we performed full waveform moment tensor inversion and waveform similarity analysis on 146 of these earthquakes. In addition, we ran a single-station detection analysis using a machine learning approach to detect 9335 earthquakes throughout the intrusion period, which allowed us to better assess the migration of the seismicity along the dike (Fig. 1G and fig. S1). The analyses of seismic and InSAR data are described in Materials and Methods and figs. S1 to S23.

## RESULTS

### InSAR and seismicity

Seismic unrest started in Fentale on 19 December 2024 at ~19:00. InSAR observations around this period (13 to 21 December 2024) showed that the intense seismicity was caused by a dike intrusion as surface motions consistent with extension across an NE-striking dike were detected on the flank of Fentale volcano. Then, seismicity migrated northward (Fig. 1G), indicating dike propagation, and InSAR observations showed extension and faulting along the central axis of the FD segment. By 3 January 2025, the dike had lengthened to ~45 km and reached Dofen, and Fentale volcano had deflated as magma flowed into the dike (Fig. 1G and fig. S3). Coeval to the dike reaching its final length, three earthquakes with magnitude *M*_w_ > 5 occurred ~20 km beyond the northern tip of the dike (Fig. 1D and fig. S1). Since then, dike lengthening stopped, but opening, seismicity, and deflation at Fentale continued at varying rates until 11 March 2025. Seismicity occurred throughout the period of dike intrusion. Analysis of the waveforms shows that earthquakes near the dike are self-similar, with the group consisting of shallow earthquakes with a mean depth of 6 ± 2 km, NW-SE–oriented tension axes, kinematics ranging from normal to strike-slip, and positive compensated linear vector dipole (CLVD) with long axes ~perpendicular to the dike (fig. S1). Substantial CLVD components, exceeding 25%, are found for the majority (~87%) of our moment tensor solutions. We assessed the CLVD uncertainties by bootstrap analysis (*22*) and found robust CLVD estimates for ~89% of the moment tensor solutions (i.e., those where the CLVD’s confidence interval are either strictly positive or negative, thus not including pure double couple solutions). These CLVD components can be explained either by complex faulting, such as the simultaneous activation of strike-slip and normal faulting, or by involving tensile components, e.g., in response to the dike intrusion. The location, timing, depth, and moment tensors of the group are consistent with dike-induced faulting, consisting of shallow normal faulting above the dike and oblique and strike-slip on faults near the dike tips (Fig. 1G and fig. S1).

**Fig. 2.**
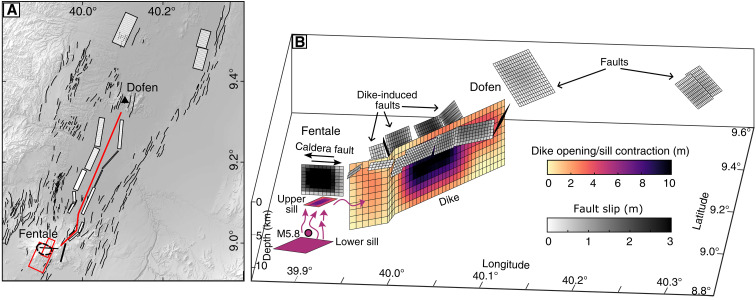
Model of the FD dike from InSAR. (**A**) Map view of the model: dike (red line), dike-induced faults (white rectangles with black outlines), and Fentale magma system: sills (red rectangles) and cross-caldera fault (black line). (**B**) Three-dimensional view of the model showing the location of the dike, faults and the two sills under Fentale. The purple arrows show magma flow, and the purple dot is the *M_w_* 5.8 earthquake (M5.8). The color bars show the total dike opening, sill contraction, and fault slip; for the kinematic model, see fig. S3.

**Fig. 3.**
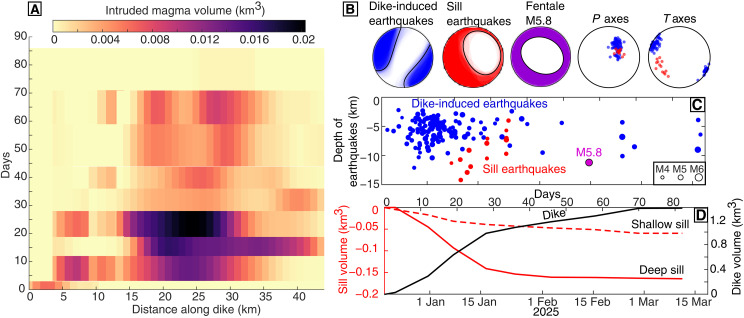
Magma volume intruded in the dike, sill volume drained, and link to seismicity. (**A**) Vertically integrated intruded magma along the dike from InSAR modeling as a function of time (days from dike onset from 19 December 2024). (**B**) Focal mechanisms of the dike-induced (blue) and sill-related (red) earthquakes. Individual mechanisms are plotted in light shade, with the most representative mechanism shown with a solid black line. The *M*_w_ 5.8 beneath Fentale is similar to the sill earthquakes, it is plotted in purple and marked M5.8. Pressure (*P*) and tension (*T*) axes from moment tensor inversion for the dike-induced earthquakes (blue) and the sill earthquakes (red). (**C**) Depth of the earthquakes from the moment tensor inversion; blue dots are the dike-induced earthquakes, and red dots are the sill earthquakes. (**D**) Cumulative volumes of dike and sills from InSAR modeling as a function of time.

To estimate the temporal and spatial distribution of the dike opening, the injected magma volume, the dike-induced faulting, and the volume decrease at Fentale, we jointly inverted CSK interferograms, CSK ROs, and S1 interferograms (Fig. 2 and figs. S3 to S14). The dike is modeled as a tensile dislocation with normal faults above it dipping toward the rift axis, and the faults correspond to structures mapped on the digital elevation model (DEM), satellite imagery, and the ROs. InSAR modeling revealed the dike reached a total volume of 1.4 km^3^ in the 3-month intrusion period, with 90% confidence interval of 1.38 to 1.45 km^3^ (Materials and Methods and figs. S15 and S16). Starting from the onset of the intrusion on 19 December, the rate of magma flow in the dike rapidly increased and peaked between 7 and 15 January to ~0.04 km^3^/day and then progressively decayed (Fig. 3A). Variable slip modeling shows that dike-induced faulting was confined to the uppermost 3 km (Fig. 2), with a total geodetic moment release of 7.3 × 10^18^ Nm. The depth and kinematics of the faulting are consistent with that derived from the moment tensor inversions. Three earthquakes with magnitude *M*_w_ > 5 also occurred ~20 km beyond the northern tip of the dike, and InSAR modeling shows that these earthquakes were caused by faulting on either side of the rift bounding faults north of Dofen (Fig. 2). Of these earthquakes, slip on two adjacent NNE-striking, east-dipping normal faults NE of Dofen has geodetically estimated magnitudes of *M*_w_ 5.6 and *M*_w_ 5.5, in agreement with the seismically recorded *M*_w_ 5.5 and *M*_w_ 5.4 on 2 and 3 of January (NEIC) (Fig. 2 and fig. S13, A to C). Slip on a third NNE-striking, east-dipping fault is modeled NW of Dofen with a geodetic magnitude of *M*_w_ 5.7 that likely corresponds to the seismically recorded *M*_w_ 5.7 on 4 of January (NEIC) (Fig. 2 and fig. S13, D to I).

The deflation of Fentale requires mainly two sources and our best-fit model consists of two stacked sills at depths of 11.6 and 5.7 km with a total volume decrease of 0.2 km^3^ (Figs. 2 and 3). Models with a single sill and a dipping dike were tested but did not fully explain the deformation pattern (Materials and Methods and figs. S17 to S19). Only the first few days of subsidence can be modeled solely by deflation of the shallowest sill, after which both sills are required (Fig. 3D). At Fentale, the moment tensor inversion and waveform similarity analysis show a prominent group of earthquakes with vertical slip kinematics and negative CLVD mechanisms (Fig. 3B and fig. S1). The earthquakes started in early January at a depth of ~10 to 15 km and, over ~2 weeks, shallowed to a depth of ~5 km (Fig. 3C). The depth range of the earthquakes is similar to the two sills modeled from InSAR data. From these observations, coupled with the earthquakes occurring when the rate of contraction of the deeper sill is highest, we interpret that the group of earthquakes is linked to especially high rates of magma withdrawal from the sills at a depth of 6 to 12 km feeding the dike (Fig. 3D). In addition, the InSAR deformation pattern could not be fully explained by the deflation sources, as horizontal motions at Fentale remained unmodeled. We therefore tested scenarios including faults on the caldera rim and/or on the flank of the volcano and found that an ~EW-striking cross-caldera fault with dominant strike-slip motion gives a best-fit (figs. S17 and S20). The fault is consistent with surface evidence and seismicity (fig. S21) (*23*–*25*), and its orientation, near perpendicular to the southern tip of the dike, and the left-lateral kinematics are consistent with stress induced by dike opening. Models with faulting at the Fentale caldera were tested but provided a worse fit (fig. S20). Motions of this cross-caldera fault occurred throughout the intrusion with no specific seismicity cluster associated with it, suggesting that the fault creeped aseismically or subseismically.

The largest earthquake in the sequence, a *M*_w_ 5.8, occurred near Fentale on 14 February, ~10 days after melt drainage from the sills decayed (Fig. 3, C and D). This earthquake occurred at a depth of ~11 ± 2 km and has a waveform and a vertical, negative CLVD mechanism similar to the other sill-related earthquakes near Fentale (Fig. 3B and fig. S1). Despite its magnitude, we do not identify any significant change in the InSAR deformation pattern, such as expected by a caldera collapse. Conversely, deep failure by such a mechanism equivalent to a *M*_w_ 5.8 at a depth of >~12 km would not cause enough deformation to be detectable by InSAR (fig. S22).

### Physics-based model of a magmatic system

The observations strongly point toward the dike being fed over several weeks from a connected magma system. The onset of diking caused depressurization first of the shallow sill (~6 km in depth), followed 3 days later by deflation of the deeper sill at a depth of about 12 km (Fig. 3D), indicating that melt connectivity was established early in the magma system and that magma pathways remained open allowing magma to flow in the dike. We can explain several key observations of the event with a relatively simple model of the magmatic system, approximated by a magma domain consisting of two sills hydraulically connected via a conduit (*26*) extending from 6 to 12 km in depth under Fentale, as suggested by InSAR modeling (Fig. 2). A detailed description of the model can be found in the Materials and Methods. The reservoir is buried in a homogeneous elastic medium and subject to extensional tectonic stress with the maximum horizontal stress parallel to the rift axis and the minimum horizontal stress perpendicular to it (fig. S2, A and B). Then, we evaluate the pressure and stress conditions as diking starts and the sills depressurize, using the analytical solution for a circular cavity under internal pressure (*27*, *28*). This model predicts that underpressure conditions occurred in the lower sill, where magma pressure became rapidly lower than the overburden, causing deep failure and large magma drainage (fig. S2B). Instead, in the upper sill magma pressure remains higher than the maximum horizontal stress for the duration of the dike (fig. S2B), meeting the condition required to keep magma pathways open, allowing melt transfer from the upper sill in the dike. The conduit connecting the sills is vertically elongated allowing it to remain open for longer than horizontally elongate reservoirs (such as sills) when the magma pressure falls below the overburden. We also evaluate the distribution of hoop stresses around the reservoir and found that our model predicts both the onset of diking at the edges of the elliptical-shaped caldera and the NE path of the dike close to Fentale, as the pressurized source locally rotates the maximum horizontal compressive stress (fig. S2C). Furthermore, we computed the stresses as magma flowed into the dike and pressure decreased in the reservoir (*28*) and found that this led to the rotation of the principal stresses. This rotation increases the shear stress on a ~EW-oriented cross-caldera fault, promoting left-lateral slip, in agreement with InSAR modeling (fig. S2, C to E). Last, we computed the reservoir depressurization at a depth of 12 km, and the associated Coulomb stresses increase onto two normal faults above the lower sill. The results show that shear failure of the reservoir was favored by the depressurization (fig. S2, G and H). The *M*_w_ 5.8 earthquake beneath Fentale occurred on 14 February when sill deflation was near to complete, and our model supports the interpretation that it is likely deep failure caused by underpressure of the deep magmatic system.

## DISCUSSION

Our results have implications for melt transfer into large dikes. Our observations and inferred models show that the 1.4-km^3^ December 2024 FD dike was intruded in a single event that lasted ~3 months and that 0.3 km^3^ of magma was sourced from the Fentale system without triggering any caldera collapse. The magma from Fentale accounts for only 16% of the injected volume but discrepancies between the volumes of magma intruded and drained are common during magma transfer (*29*, *30*). During the 2005 Dabbahu intrusion, the contraction of the magma chambers only account for 20% of the magma in the dike (*2*), comparable to Fentale. Discrepancies have been explained by magma compressibility. If the magma remaining in the chamber contains exsolved gases, then it will expand when the pressure drops, and the volume change of the magma chamber will be estimated to be many times smaller than the volume of magma withdrawn (*29*). Therefore, it is possible that all the injected magma came from the Fentale reservoirs. Another explanation is that additional magma came from deeper than our 12-km-depth sill. A source at these depths would not be visible in the InSAR. The December 2024 FD dike was the second most voluminous dike observed geodetically after the 2005 Dabbahu-Manda Hararo dike (~2.5 km^3^) of Afar (*2*, *3*). It was preceded shortly by another dike in September 2024 (*21*), marking the beginning of a rifting episode (*1*), similar to the recent processes in Iceland (*5*, *31*).

It is well established that in extensional tectonic settings dikes relieve tensional stresses built up by the long-term tectonic extension (*1*, *4*, *5*). This explains why the initial intrusion of a rifting episode is typically the most voluminous, while eruptions are small or absent until the extensional stresses are reduced by repeated intrusions. Some new ideas have been suggested that high tension and large magma availability are enough to trigger dike formation such as in Iceland and Hawaii (*5*, *32*–*33*). The FD dike propagated ~45 km in the crust and was accompanied by faulting without any eruption, indicating that tensile stresses were high and yet to be fully relieved. In addition, large volumes of magma must have accumulated under Fentale for some time before the December 2024 dike. InSAR shows modest inflation over ~3.5 years, from January 2021 to September 2024, consistent with a sill at a depth of 12 km and a total volume increase of ~0.02 km^3^ (fig. S23), only a small fraction of the 1.4-km^3^ intruded magma. Despite high tensile stress and magma availability, all recent previous dikes in FD (the September 2024 and in 2015) are over an order of magnitude smaller than the December 2024 dike (*20*, *21*). Furthermore, during these early intrusions, no melt transport from the deep magma storage system was observed. This is best explained by an initial lack of connectivity between discrete reservoirs in the plumbing system. We propose the September 2024 dike caused fracturing and opened new pathways that increased magma connectivity of the storage system between 6 and 12 km in depth. This, jointly with the development of underpressure and failure of the deep reservoir, created the conditions for draining substantial magma volumes. This model is commonly challenged by the suggestion that magma pathways will close upon underpressure, hence inhibiting magma flowing from a deep reservoir (*34*). However, we provide a physical model that can reconcile this discrepancy, owing to the presence of a connected magma system of sills and a conduit. Our calculations of stresses and pressure changes show that the deeper sill, an horizontally elongated reservoir, will develop underpressure and collapse before the connecting vertical conduit (fig. S2B). This can explain how vertical magma pathways remain open allowing magma to flow despite deep underpressure.

During the 2014–2015 Bardarbunga rifting event, underpressure drove large magma drainage from a ~5-km-depth reservoir, and caldera collapse followed (*4*). The Bardabunga caldera has a diameter of 10 km, compatible with a caldera-supporting reservoir ~5 km in depth (*35*–*37*) as the source of magma underpressure. Instead, the diameter of the Fentale caldera is only ~5 km consistent with a caldera-forming reservoir at a depth of ~2.5 km and therefore much shallower than our deflating shallow sill at a depth of 6 km. We suggest that the Fentale caldera did not collapse because the shallow sill at a depth of 6 km is not the caldera supporting reservoir and shallower sills likely exist but remained disconnected.

Our results are the first to show that large volume dikes can be intruded only when magma storage connectivity is established and underpressure develops, triggering deep-seated failure and creating the conditions to drain large volumes of magma. Only then, the large volumes of available magma can flow into areas of high tensional stress. The interpretation is globally important since it provides an insight into the architecture of magmatic systems that likely supply the giant dike swarms of Earth commonly associated with flood basalts and subsequent continental breakup [e.g. (*38**–**39*)]. A vertically extensive magmatic system provides an explanation to accumulate large volumes of melt in the prediking period with insufficient overpressure for triggering reservoir failure and also explains the slow pressure drop needed for protracted intrusion of large dikes (*40*). In addition, the depth extent of the system allows storage of buoyant melt in the deep crust, where the ductile rheology further aids accumulation of large magma volumes but without causing substantial ground surface motions (*40*). Because of the challenges in observing deep crustal magma motions with geodetic and geophysical techniques, deep connected magma systems may be more common in continental rifts than commonly interpreted. At Fentale, it was the connected and underpressurized deep magmatic storage that allowed large volumes of magma flow into the dike and caused detectable seismic and geodetic signals.

## MATERIALS AND METHODS

### Interferometric Synthetic Aperture Radar

We generated interferograms using C band SAR radar data from Sentinel-1A (S1) satellite of the Copernicus program (European Space Agency) in descending (track 079) and ascending (track 087) geometries (table S1). We also generated interferograms using X band SAR data from CSK constellation (Italian Space Agency) in ascending (track HI-02) and descending (tracks HI-04 and HI-22) geometries (table S1). The S1 SAR images are acquired through the Terrain Observation with Progressive Scans Interferometric Wide Swath acquisition mode, which provides a ~250 km wide scene. S1 acquisitions over Fentale were relatively limited during the dike intrusion so we also used CSK data acquired in StripMap mode to complement the S1. The CSK data are provided as scenes with a width of ~40 km and a length of 50 km. Each scene was processed as raw data (level 0) and then concatenated and merged to provide ~100-km-long final interferograms. The S1 and CSK interferograms were processed with the InSAR Scientific Computing Environment (ISCE2) software package (*41*). We coregistered the Single Look Complexes (SLCs) and removed the topographic phase using a 1–arc sec (∼30-m resolution) Shuttle Radar Topography Mission (SRTM) DEM. We then filtered the interferograms using a Goldstein adaptive power spectral filter with a strength of 0.5 (*42*). Last, we unwrapped the interferograms using the branch cut algorithm and geocoded them using the 1–arc sec SRTM DEM.

We also applied the Pixel Offset Tracking technique to overcome the lack of data in the interferograms in areas characterized by of high deformation gradient where phase unwrapping could not be resolved and coherence was lost (*43*). We generated RO displacement maps for ascending (track HI-02) and descending (tracks HI-04 and HI-22) CSK data pairs (table S1) using the technique described in (*44*) and references therein. Cross-correlation was computed on a 64-by-64 pixels matching window, by considering all the pixels across both range and azimuth directions. Starting from the full-resolution SAR image grid, the RO estimates are converted into meters and geocoded through a nearest-neighbor method to the 1–arc sec SRTM DEM grid, thus directly comparing the RO measurements with InSAR interferograms. RO accuracy is on the order of ^1^/_10_ of the pixel size of the amplitude SAR images (*44*), which corresponds to about 0.3 m along the range direction in the case of CSK StripMap data.

For the InSAR modeling, we assumed shear and tensile dislocations in a uniform elastic half space with a Poisson’s ratio of 0.25 and a shear modulus of 30 GPa. The dike was modeled as a vertical tensile dislocation, and the faults above the dike were modeled as shear dislocations dipping toward the rift axis. The magma plumbing at Fentale was modeled with two horizontal tensile dislocations, approximating two sill-shaped magma chambers, and a ~EW-striking fault cross-cutting the caldera. The best-fit parameters of the sources were initially inferred using a nonlinear Monte Carlo simulated annealing inversion (*45*), assuming uniform dike opening and fault slips. Before running the inversion, all interferograms were quad-tree partitioned on the basis of the deformation gradient to reduce the data size. At least two interferograms, one ascending and one descending, were jointly inverted for each period of the dike intrusion (figs. S4 to S13). The interferograms were weighted by the inverse of the data error variance. This was estimated using the squares of the root mean square (RMS) misfit from the inversion of each individual interferogram. Then, we carried out a least-squares inversion to determine the spatially variable dike opening, fault slip, and contraction of the magma chambers, keeping fixed the model geometry from the uniform models. To prevent unphysical oscillatory solutions, Laplacian smoothing was imposed. The dislocation planes were discretized into patches of 0.5 km by 0.5 km for sills and faults and 1 km by 1 km for the dike. For the best-fit models, we selected a smoothing factor that minimizes the trade-off between the model RMS misfit and solution roughness (fig. S14). We use a single smoothing factor for all the dislocations, and we choose relatively unsmoothed solutions for all time intervals to minimize oversmoothing.

We started with the nonlinear inversion of each set of interferograms for each time period. Initially, we used dike sources only and left all search bounds free to vary. Then, we tightly constrained the geometry of dike segments and added the faults. We used one dike segment for the initial 6 to 22 December 2024 period and three dike segments for time periods afterward. We used different numbers of faults for the different time periods, as these were not all active simultaneously but slipped as the dike lengthened. We fixed the number and location of faults above the dike using optical imagery and the ROs. The faults above the dike were assumed to be normal. For the Fentale volcano, we used one shallow sill for the 6 to 22 December 2024 period and two sills and a transverse fault for time periods afterward, albeit other source combinations were tested (see description below). All the other model parameters were left free to vary in the nonlinear inversion. The triggered faults, ~20 km north of Dofen were inverted separately, and all parameters were allowed to vary. In the least-squares inversion to determine the spatially variable dike opening, fault slip, and contraction of the magma chambers, we used only a shallow sill and a dike segment for the 6 to 22 December 2024 period. For all time periods afterward, all sources were included leaving the linear inversion free to find fault slips and dike opening for all time steps.

Uncertainties on the model parameters of the nonlinear inversion were estimated by simulating 100 sets of spatially uncorrelated random Gaussian noise with the same variance function as observed in the InSAR data. The noise was added back to the original data to obtain a noisy dataset that was then inverted to produce 100 best-fit model parameters. For this inversion, we kept fixed the fault locations and the length and width of the dike segments while leaving the dike opening and fault slip free to vary. The distribution of the best-fit solutions determines the parameters uncertainties and shows that the dike volume range of the models falls in a narrow interval for all the separate time steps. The total dike volume, 1.4 km^3^, has a 90% confidence interval between 1.38 and 1.45 km^3^ (fig. S15). In addition, trade-offs between the dike volume and the fault slips were investigated for each time interval (fig. S16). Some trade-offs are evident, but, overall, most of the fault slip solutions fall in a narrow range.

For Fentale, we tested different combinations of deformation sources. In fig. S17, we show the best-fit models assuming a single sill, two sills, and two sills with a cross-caldera fault. For the beginning of the intrusion, 6 to 22 December 2024, we tested a model with a vertical or a dipping dike without the shallow sill (fig. S18), but this model provides a worse fit to the data compared to the model with a shallow sill, indicating that this reservoir was active from the beginning of the intrusion. The presence of the shallow sill under Fentale is also confirmed by the postintrusive deformation. Fentale has been inflating continuously since the end of the intrusion. We model the postintrusive inflation for two separate posteruptive time periods, 22 February 2025 to 27 June 2025 and 31 November 2025 to 29 January 2026, and found that, for both periods, the inflation is best explained by a shallow sill with similar location and depth (6 to 7 km in depth) as the cointrusive sill (fig. S19). Last, in fig. S20, we show the best-fit models assuming faulting on the Fentale caldera, both normal or reverse, and a cross-caldera fault. For this test, we specifically selected a short time interval in early February (3 to 15 February 2026) when the deflation of the two sills decreased and the effect of faulting is more clearly visible. The modeling results show that our preferred model of two sills and a cross-caldera fault fit the data better than the other models.

### Seismicity

To relocate the seismicity (fig. S1), we ran a single-station detection analysis using a machine learning–based approach (*46*) to detect P and S phases and associated peaks whenever differential S-P times fall in the interval of 13 to 25 s (most of our dike-induced earthquakes have differential times of 15.5 to 22.5 s). We used a detection threshold of 0.3 for P and 0.1 for S and performed the analysis on raw data after applying a bandpass filter in the range of 0.05 to 1 Hz. Processing continuous data from the two sensors (channel codes 00 and 10) produced qualitatively similar results in terms of differential times, with both cases finding a median differential time of ~18.5 s. However, data from the sensor code 10 outperformed those from sensor code 00 in terms of detections (9335 detections versus 6727). With the assumption of the earthquakes having similar shallow depths and locations near the FD segment, as indicated by the InSAR modeling, we associated the single-station differential times to locations along FD and thus reconstructed the temporal evolution of the events along the dike from its original starting point (Fig. 1D). We also performed a full waveform deviatoric moment tensor inversion, fitting three component displacement seismograms in the vertical, radial, and transversal components in the time domain. We considered open broadband seismic stations up to 2000 km away. The inversion was performed using the probabilistic source inversion algorithm Grond (*22*). Local data, with epicentral distances of <250 km, are modeled using a local velocity model (*47*). At regional distances between 250 and 500 km and further distances between 250 and 2000 km, we relied on a regional model (*48*) and a global AK135 model (*49*), respectively. We resolved centroid location, centroid depth, centroid time, and deviatoric moment tensor components for 146 events. The mean deviatoric moment tensors were then grouped in two main families, according to their moment tensor geometry, using cluster analysis of the focal mechanisms with Seiscloud (*50*) (fig. S1). We independently tested the cluster analysis by calculating the waveform similarity at station FURI and found that the two groups of earthquakes are similar to the cluster analysis, supporting the results of moment tensor clustering. This illustrates how dike-related seismicity is predominant in the early phase, while reservoir-related seismicity starts after the dike reached Dofan.

### Physical model of magmatic system

We propose a model of the deep magmatic storage with two sills, as inferred from the InSAR. The sills are melt-dominated zones at depths of 5 and 12 km and are hydraulically connected via a conduit that we envisage corresponds to crystal mush (fig. S2A). The reservoir is buried in a homogeneous elastic medium and subject to extensional tectonic stress with the maximum horizontal stress, $S_{H}$, parallel to the rift axis and the minimum horizontal stress perpendicular, $S_{h}$, to it (fig. S2, A and B). We assume that the pressure in the lower sill at a depth of 12 km is initially slightly above lithostatic. Using a host rock density, $\rho_{C}$, of 2890 kg/m^3^ and a magma density, $\rho_{f}$, of 2100 kg/m^3^, we calculate the initial (prediking) pressure within the reservoir as $P_{\left(t=t_{1}\right)}=\left(\rho_{C}-\rho_{f}\right)\cdot g\cdot \left(z_{12}-z\right)$ (fig. S2B). As the diking begins, the upper sill drains and depressurizes first, followed by depressurization of the connecting conduit and the lower sill. Then, we evaluate the pressure and stress conditions using the analytical solution for a circular cavity under internal pressure, P; this is a widely used approach to modeling dikes near volcanoes (*26*–*28*). This model predicts that underpressure conditions first occurred in the lower sill, where magma pressure became rapidly lower than the overburden, $S_{V}$, causing deep failure and large magma drainage (Fig. 2B). Underpressure conditions will migrate upward but progressively. The bright red segment in fig. S2B, representing the magma pressure within the system, moves to the left maintaining its slope, and it will cross the line of the horizontal stress (pink line in fig. S2B, representing the stress component normal to the conduit) at progressively shallower levels. Instead, in the upper sill, magma pressure remains higher than the maximum horizontal vertical stress for the duration of the dike (Fig. 2B). This ensures meeting the condition required to keep the magma pathways open, allowing melt transfer from the upper sill in the dike. The conduit connecting the sills is vertically elongated, allowing it to remain open for longer than horizontally elongate reservoirs (such as sills) when the magma pressure falls below the overburden. We also estimated the hoop stress, $\sigma_{\Theta \Theta }$, at the margin of the reservoir to determine where this stress becomes tensile (>0), hence triggering a dike. We assumed a size and shape of our reservoir similar to Fentale, a Poisson ratio of 0.25, and a shear modulus of 30 GPa (*28*). We find that the trigger condition occurs when the reservoir overpressure reaches about half of the tectonic stress, $S_{H}$, at a depth of 5 km (fig. S2F). The hoop stress becomes positive in the direction parallel to the elongated end of the caldera (at an angle with the rift axis of 90°; fig. S2F), in agreement to where diking started, on the eastern side of Fentale. We also show that the trajectory of the Fentale dike can be predicted using this solution (fig. S2C).

Local stress conditions near the Fentale caldera are modified by the evolving pressure within the reservoir (*28*). As diking starts and the reservoir depressurizes, the principal stresses tend to rotate according to the extensional stress regime (fig. S2D). This rotation increases the shear stress on a ~EW-oriented cross-caldera fault, promoting left-lateral slip. The slip rate is expected to correlate closely with the rate of sill drainage and dike injection, as modeled by InSAR (fig. S2E). Last, we estimated the Coulomb stress change at a depth of 12 km as the reservoir was depressurized and showed that the Coulomb stress increased close to Fentale (fig. S2, G and H), promoting deep normal faulting earthquakes such as the *M*_w_ 5.8 earthquake on the 14 February 2025 when the lower sill was depressurized below lithostatic conditions.
