# Supplementary material for: Deep magma underpressure and connectivity drive large dike intrusions
Source: Sci Adv. 2026 Jul 8;12(28):eaed6073. doi: 10.1126/sciadv.aed6073 (PMC13344291; doi:10.1126/sciadv.aed6073)
Supplement: Supplementary file 1 — Figs. S1 to S23 Table S1 [file sciadv.aed6073_sm.pdf]

Science Advances
AAAS

Supplementary Materials for

Deep magma underpressure and connectivity drive large dike intrusions

Carolina Pagli *et al.*

Corresponding author: Carolina Pagli, carolina.pagli@unipi.it

Sci. Adv. **12**, eaed6073 (2026)
DOI: 10.1126/sciadv.aed6073

This PDF file includes:

Figs. S1 to S23
Table S1

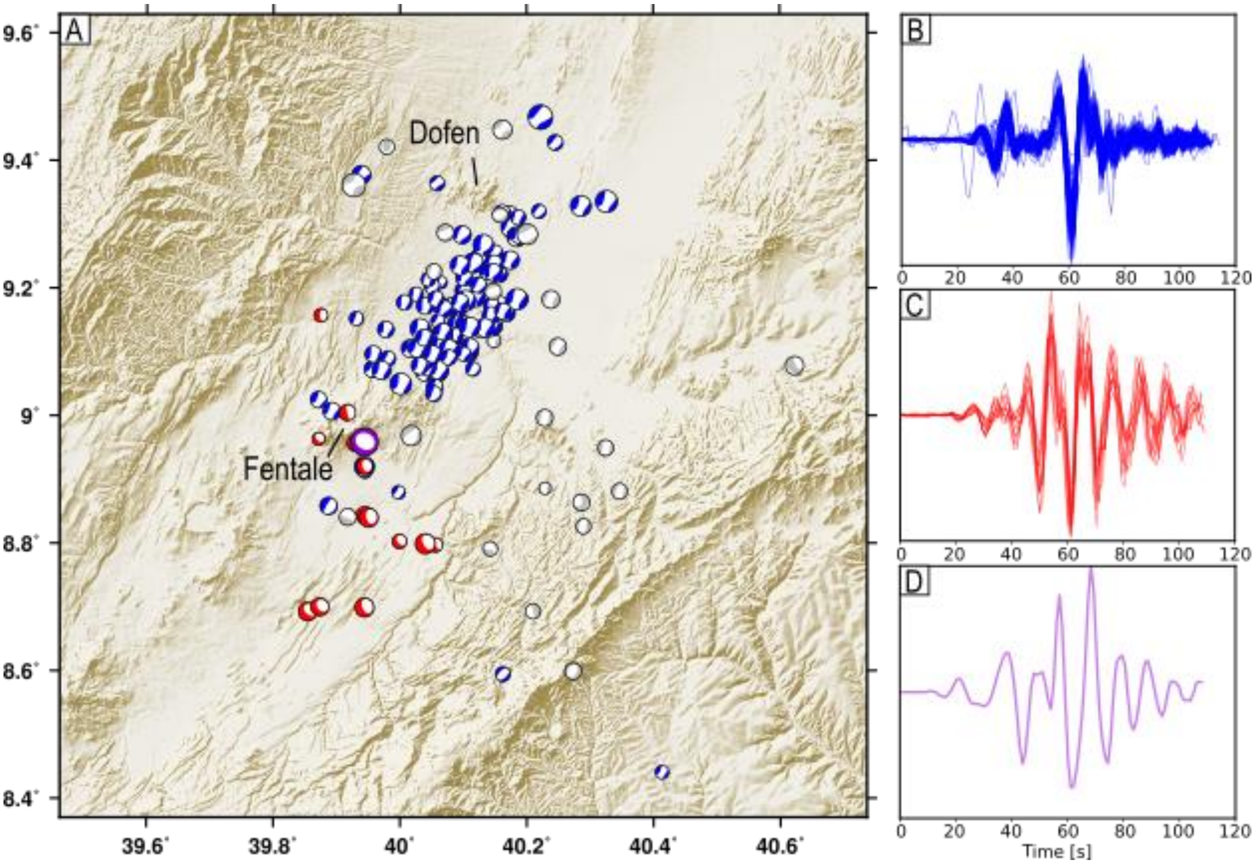

Fig. S1. Seismicity. (**A**) map of computed locations and moment tensors with the colors corresponding to similar mechanisms and to the plots in (**B**), (**C**), and (**D**) of the similar waveforms in the main earthquake groups. Blue is for the dike-induced earthquakes, red for the sill-contraction related earthquake near Fentale, purple is for the M_w5.8 earthquake below Fentale, and gray is used for outliers in the moment tensor distribution, often corresponding to weak events with scattered locations and/or more noisy waveforms.

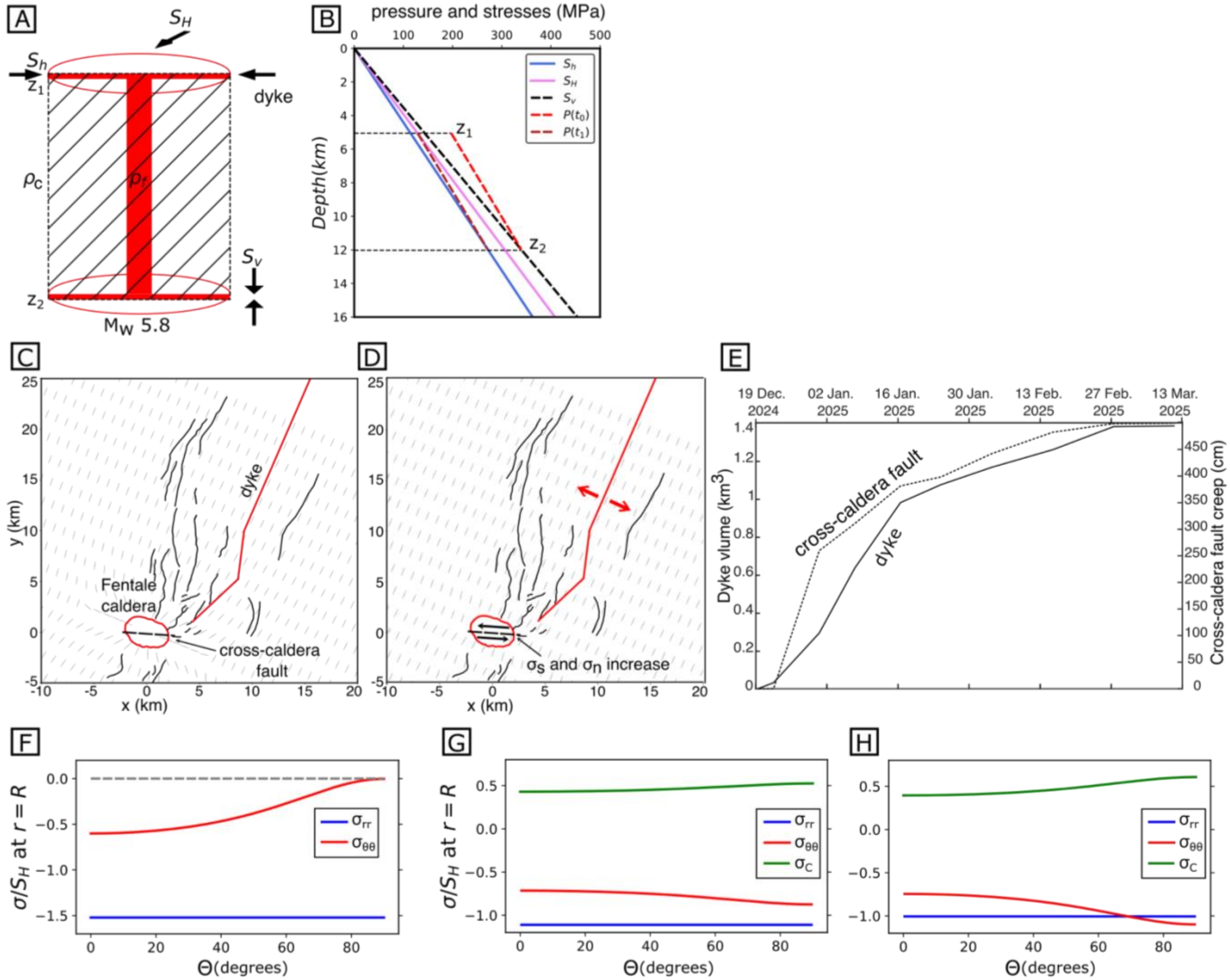

Fig. S2. Physical model of transcrustal magmatic system. (**A**) sketch of the model, z_1 is the depth of the upper sill, 5 km, and z_2 is the depth of the lower sill, 12 km. The onset of diking at the eastern end of the caldera is marked, and the M_W 5.8 at the depth of the lower sill is marked. (**B**) Magma pressure, *P*, and stresses with depth calculated at the onset of diking at time t_0 and at the end of diking at time t_1. (**C**) Directions of maximum compressive stress S_H (grey dashes) at 5 km depth at onset of diking and (**D**) at the end of diking. (**E**) Cumulative intruded volume of dike (solid black line) and cross-caldera fault creep (dashed line) from InSAR modelling as a function of time. (**F**) Circumferential hoop stress, $\sigma_{\Theta\Theta}$, and radial hoop stress, σ_{rr}, at the margin of the reservoir as a function of the angle with the rift axis, Θ. Compressional stress is taken negative. The hoop stress is positive and tensile stress occurs at the angle $\Theta = 90°$. (**G**) Coulomb stress changes, σ_c, at dike onset on normal faults at 12 km depth (green lines) as a function of the angle with the rift axis, Θ. Circumferential hoop stress, $\sigma_{\Theta\Theta}$, and radial hoop stress, σ_{rr}. The Coulomb stress is almost constant and $\sim 0.5 \cdot S_H$. (**H**) Coulomb stress changes as pressure in the reservoir drops. The Coulomb stress increases to $\sim 0.7 \cdot S_H$.

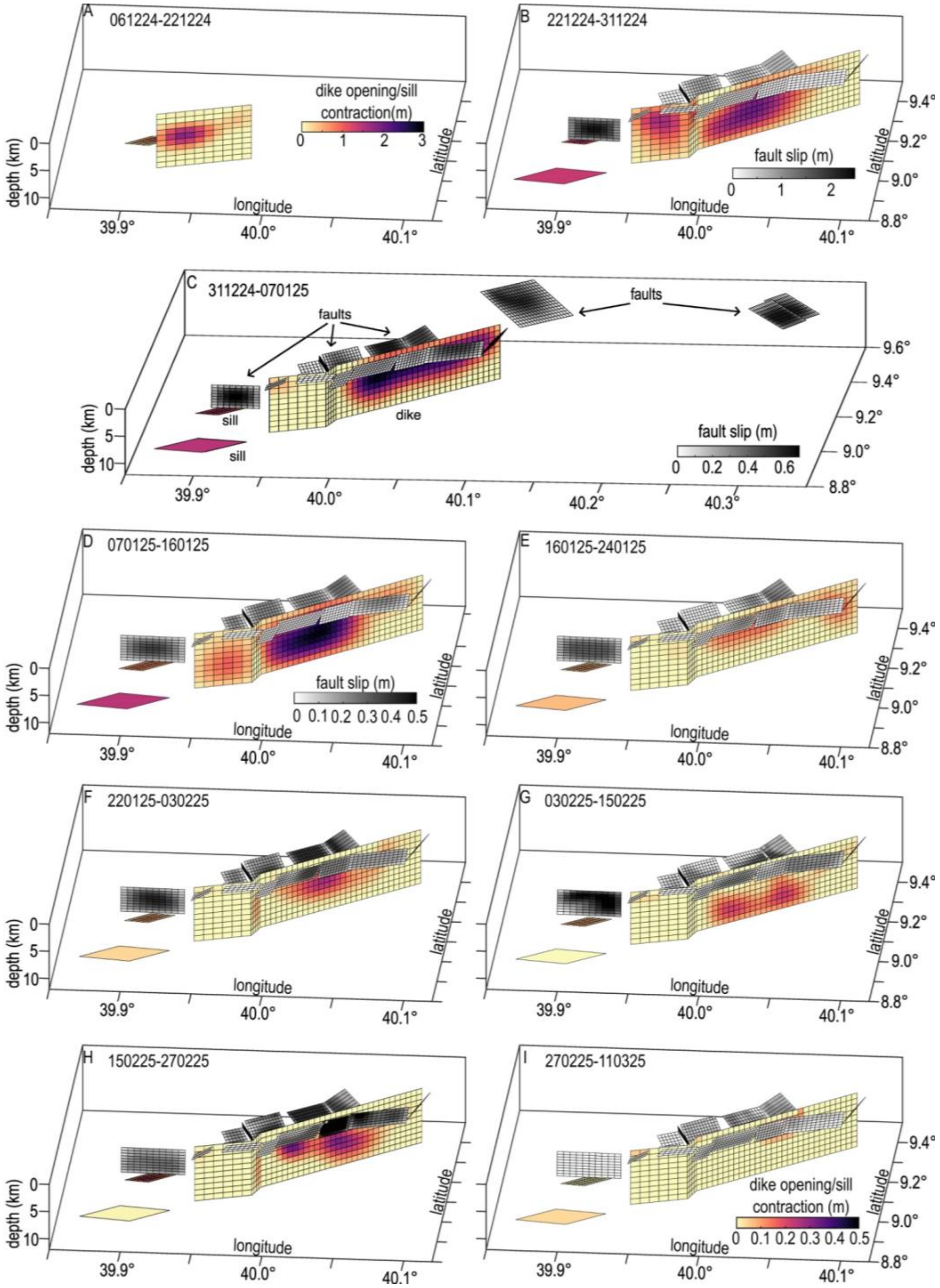

Fig. S3. Kinematic InSAR modelling. Temporal evolution of the dike intrusion, fault slips and sill contraction from InSAR inversion, for the plan view see Fig. 2A. In the top left corner are the time intervals as ddmmyy. The colorbar of dike opening and sill contraction in (**A**) also applies to panels (**B-H**), the colorbar of fault slip in (**D**) also applies to (**E-I**). Observed, modeled and residual InSAR for each time interval are in Fig. S4-13.

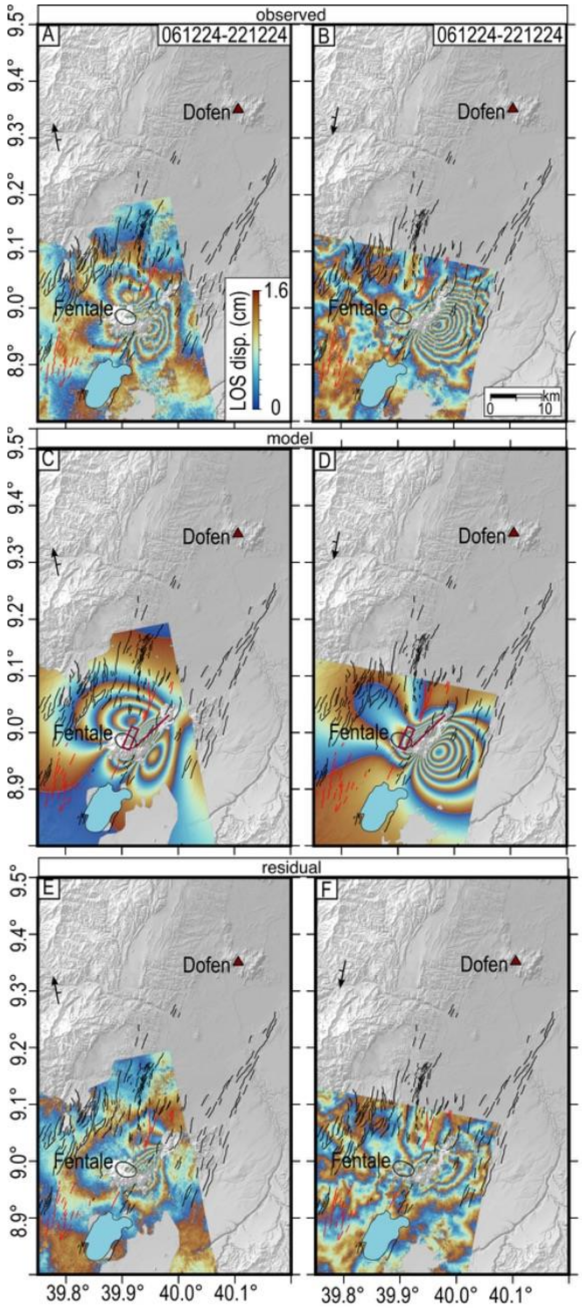

Fig. S4. InSAR modelling of 6-22 December 2024. (**A-B**) observed CSK ascending and descending interferograms. In the top right corner are the time intervals as ddmmyy. (**C-D**) modelled CSK interferograms. (**E-F**) residual CSK interferograms. The red rectangle is the sill and red line is the dike as in Fig. S3A. The values in the interferograms are in the satellite line-of-sight (LOS) direction and positive values mean a range increase (motion away from the satellite), such as caused by deflation. The satellite orbit and the LOS are shown by the black arrow with the tick.

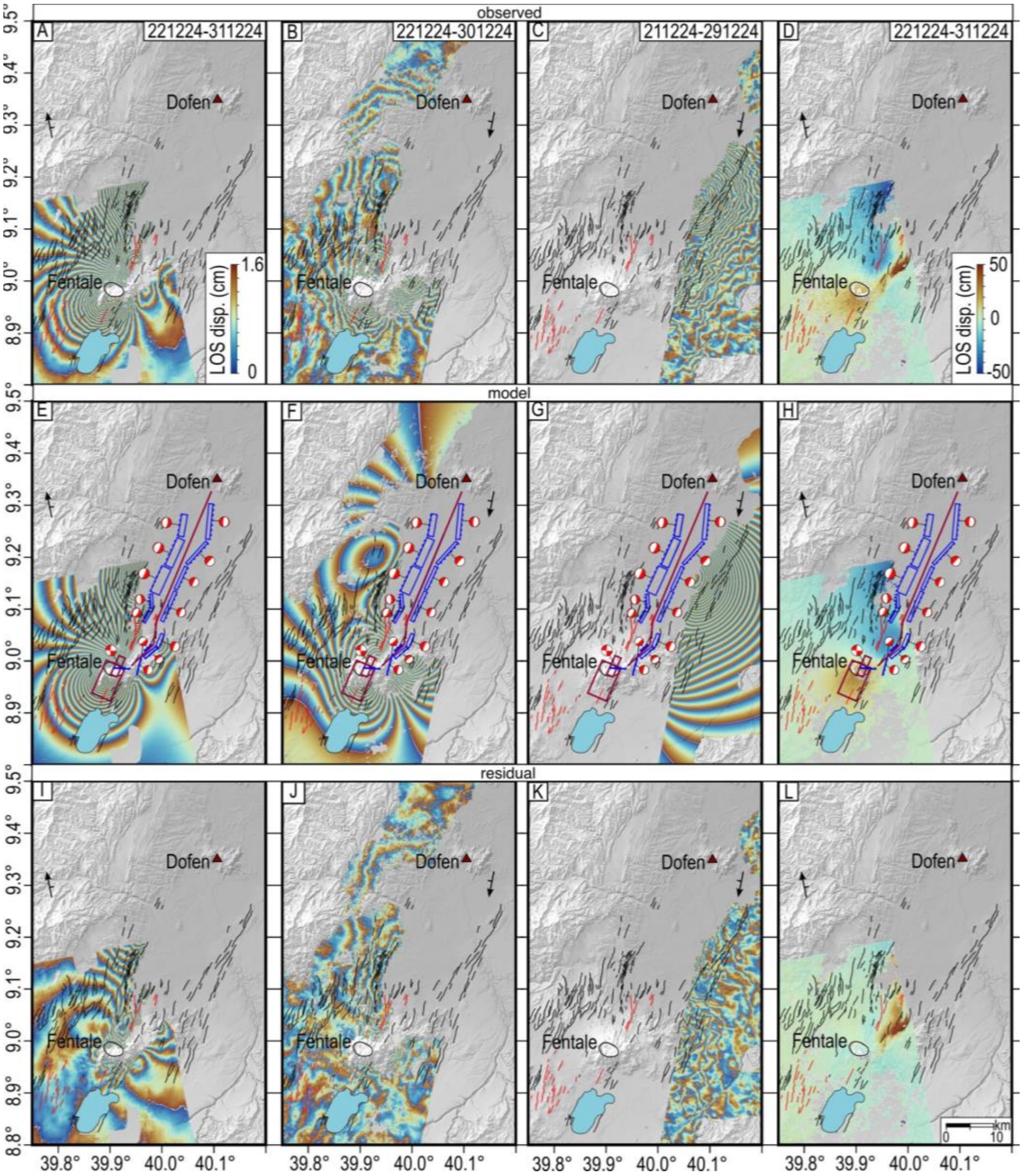

Fig. S5. InSAR modelling of 22 December 2024-31 December 2024. (**A-D**) observed CSK ascending and descending interferograms, and range offset. In the top right corner are the time intervals as ddmmyy. (**E-H**) modelled CSK interferograms, and range offset. The red rectangles are the sills, the red line is the dike and the blue rectangles are the faults as in Fig. S3B. The red beach-balls are the focal mechanisms from the inversion. (**I-L**) residual CSK interferograms, and range offset. The values in the interferograms are in the satellite line-of-sight (LOS) direction and positive values mean a range increase (motion away from the satellite), such as caused by deflation. The satellite orbit and the LOS are shown by the black arrow with the tick.

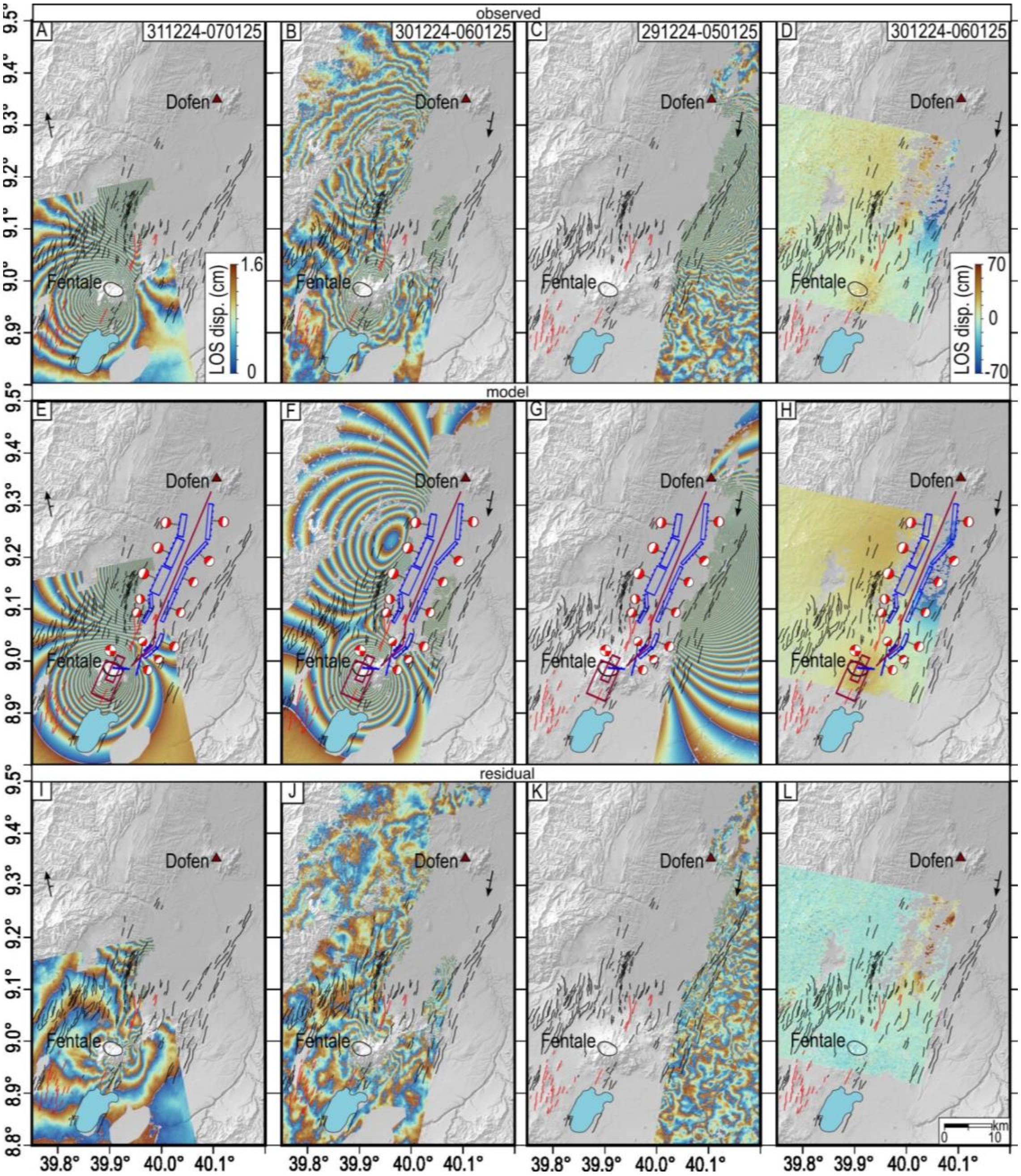

Fig. S6. InSAR modelling of 29 December 2024-07 January 2025. (**A-D**) observed CSK ascending and descending interferograms and range offset. In the top right corner are the time intervals as ddmmyy. (**E-H**) modelled CSK ascending and descending interferograms, and range offset. The red rectangles are the sills, the red line is the dike and the blue rectangles are the faults as in Fig. S3C. The red beach-balls are the focal mechanisms from the inversion. (**I-L**) residual CSK ascending and descending interferograms, and range offset. The values in the interferograms are in the satellite line-of-sight (LOS) direction and positive values mean a range increase (motion away from the satellite), such as caused by deflation. The satellite orbit and the LOS are shown by the black arrow with the tick.

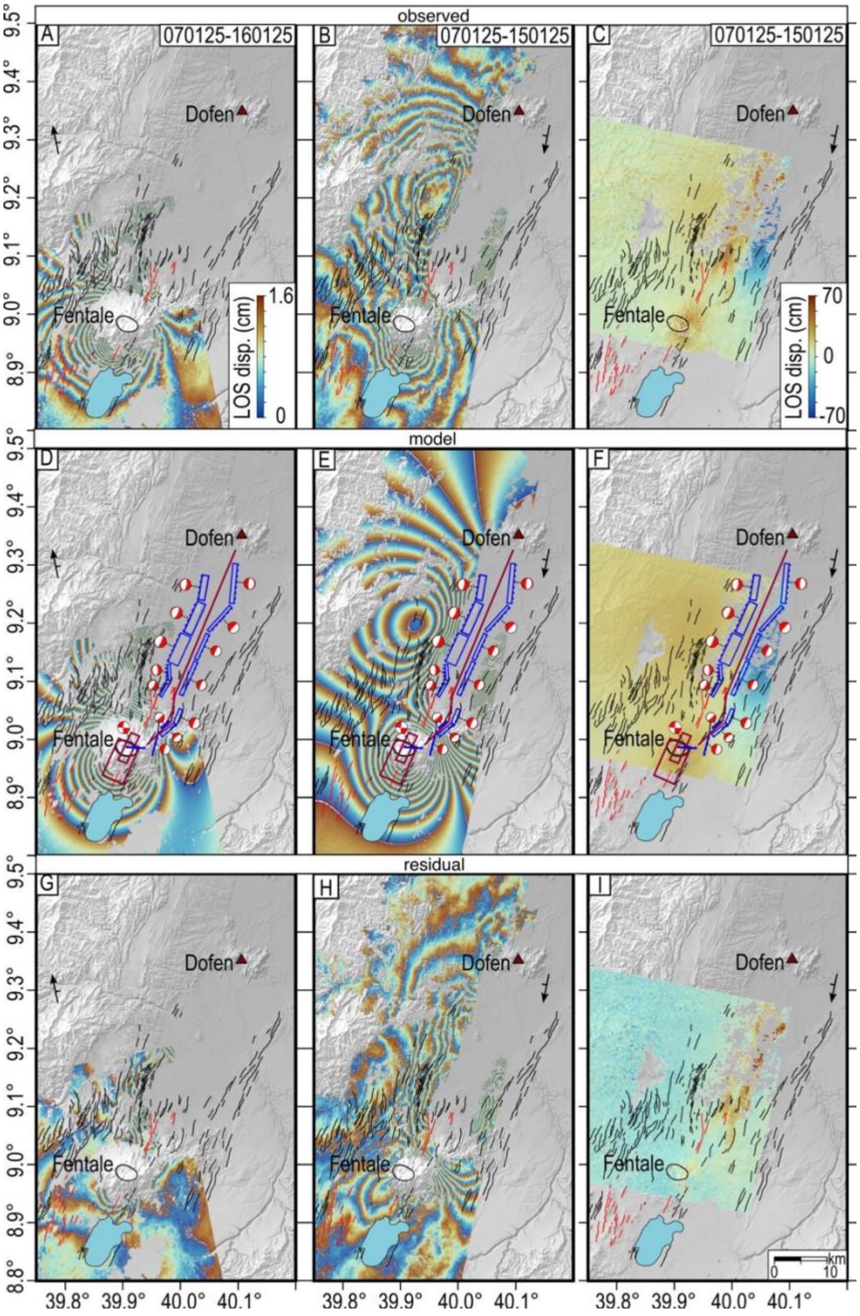

Fig. S7. **InSAR modelling of 07-16 January** 2025. (**A-C**) observed CSK ascending and descending interferograms, and range offset. In the top right corner are the time intervals as ddmmyy. (**D-F**) modelled CSK ascending and descending interferograms, and range offset. The red rectangles are the sills, the red line is the dike and the blue rectangles are the faults as in Fig. S3D. The red beach-balls are the focal mechanisms from the inversion. (**G-I**) residual CSK ascending and descending interferograms, and range offset. The values in the interferograms are in the satellite line-of-sight (LOS) direction and positive values mean a range increase (motion away from the satellite), such as caused by deflation. The satellite orbit and the LOS are shown by the black arrow with the tick.

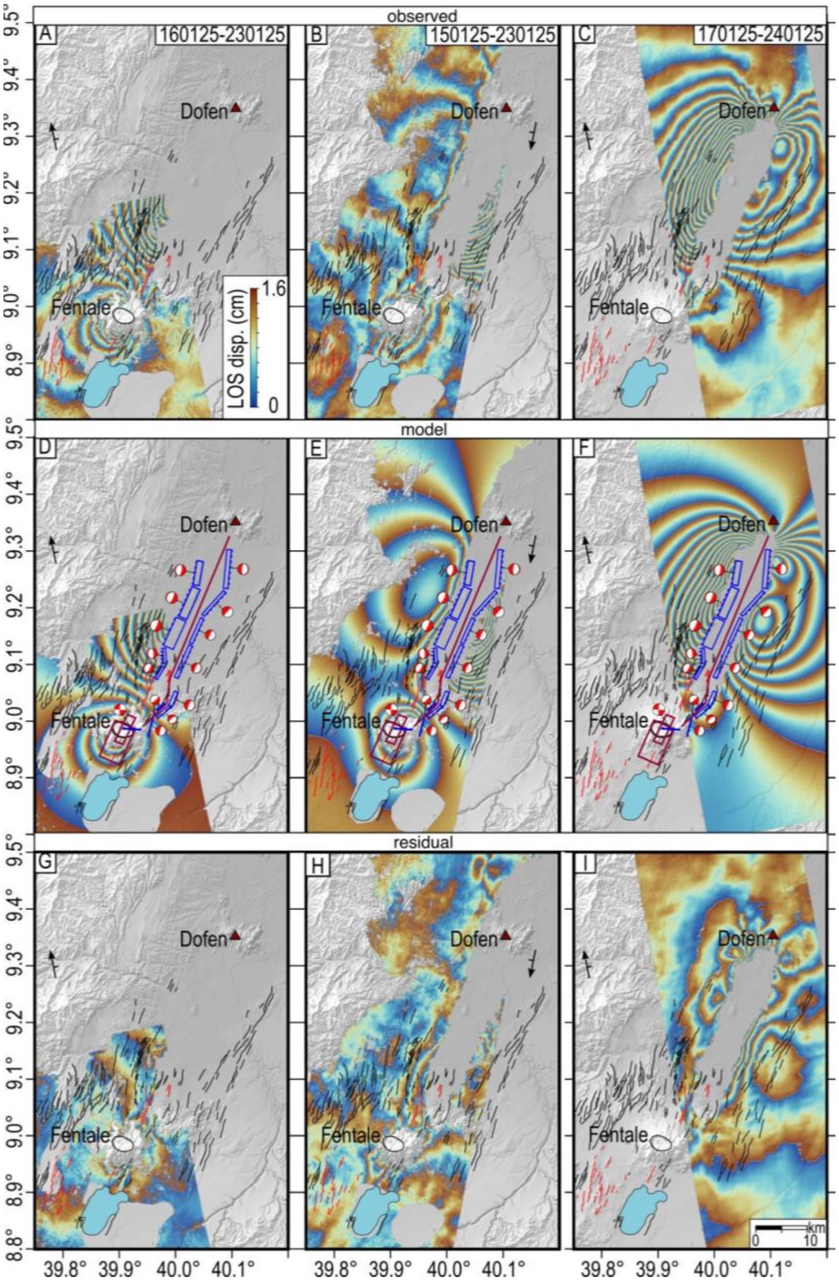

Fig. S8. InSAR modelling of 15-24 January 2025. (**A-C**) observed CSK ascending and descending interferograms. In the top right corner are the time intervals as ddmmyy. (**D-F**) modelled ascending and descending CSK interferograms and modelled CSK range offset. The red rectangles are the sills, the red line is the dike and the blue rectangles are the faults as in Fig. S3E. The red beach-balls are the focal mechanisms from the inversion. (**G-I**) residual ascending and descending CSK interferograms and modelled CSK range offset. The values in the interferograms are in the satellite line-of-sight (LOS) direction and positive values mean a range increase (motion away from the satellite), such as caused by deflation. The satellite orbit and the LOS are shown by the black arrow with the tick.

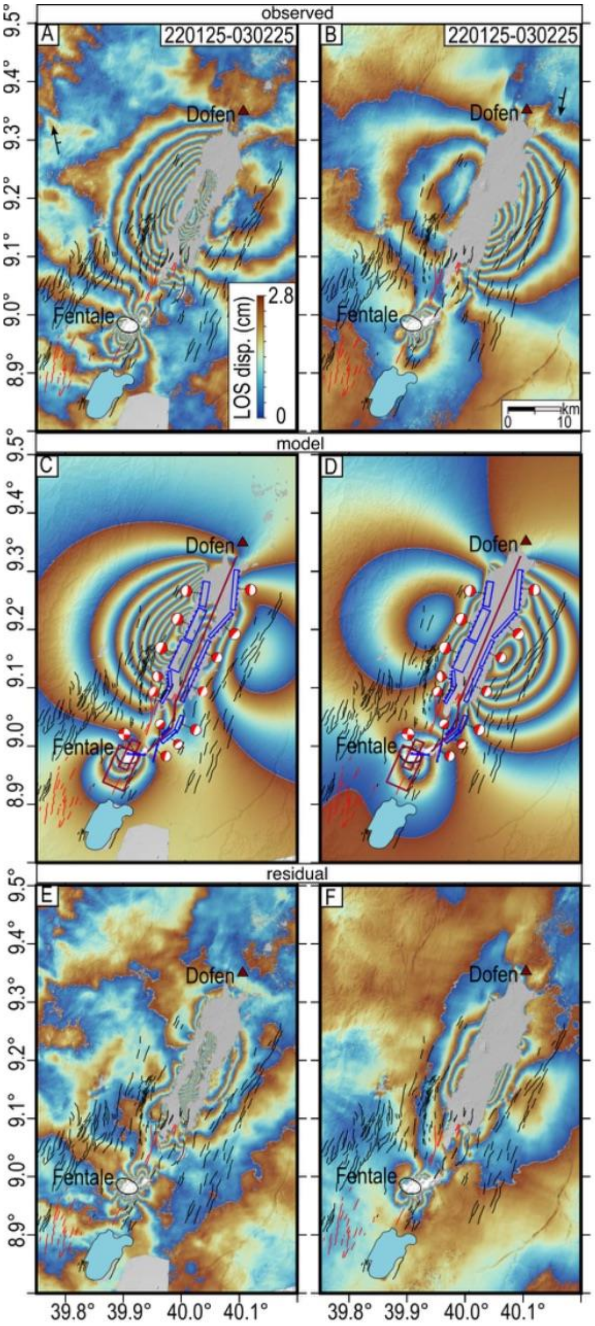

Fig. S9. InSAR modelling of 22 January 2025-03 February 2025. (**A-B**) observed ascending and descending S1 interferograms. In the top right corner are the time intervals as ddmmyy. (**C-D**) modelled S1 interferograms. The red rectangles are the sills, the red line is the dike and the blue rectangles are the faults as in Fig. S3F. The red beach-balls are the focal mechanisms from the inversion. (**E-F**) residual ascending and descending S1 interferograms. The values in the interferograms are in the satellite line-of-sight (LOS) direction and positive values mean a range increase (motion away from the satellite), such as caused by deflation. The satellite orbit and the LOS are shown by the black arrow with the tick.

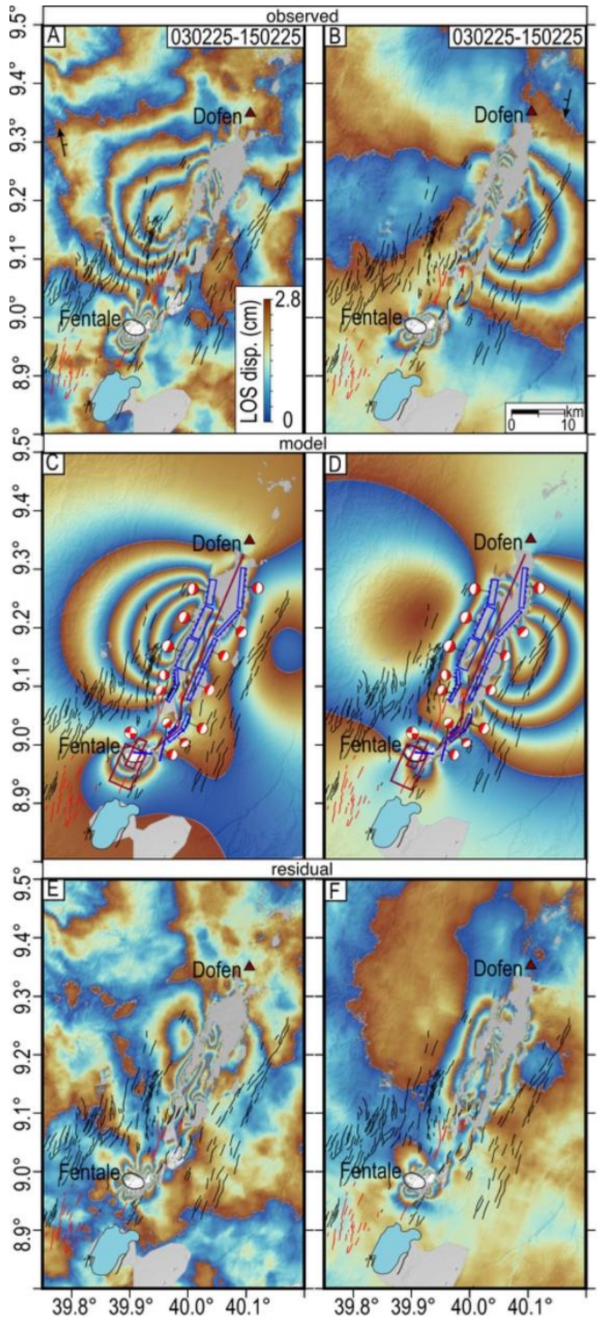

Fig. S10. Modelling of S1 interferograms spanning the period 03 February 2025-15 February 2025. (**A-B**) observed ascending and descending S1 interferograms. In the top right corner are the time intervals as ddmmyy. (**C-D**) modelled ascending and descending S1 interferograms. The red rectangles are the sills, the red line is the dike and the blue rectangles are the faults as in Fig. S1G. The red beach-balls are the focal mechanisms from the inversion. (**E-F**) residual ascending and descending S1 interferograms. The black arrow gives the satellite orbit trajectory with the black tick marking the viewing geometry.

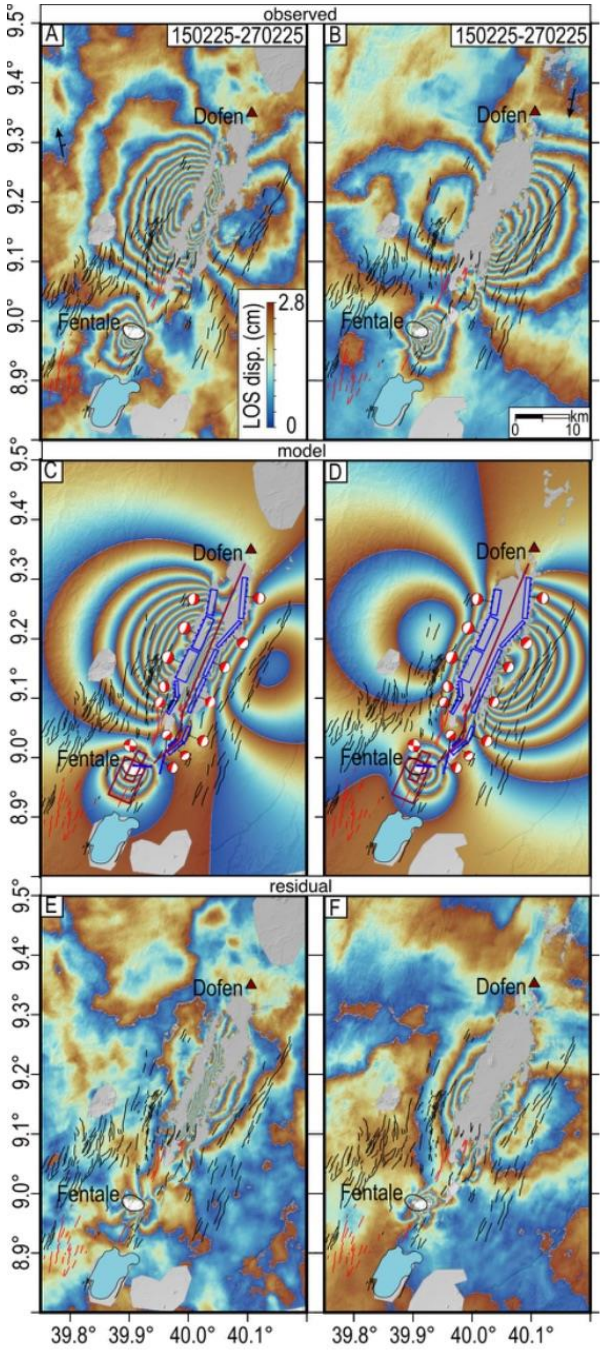

Fig. S11. InSAR modelling of 5-27 February 2025. (**A-B**) observed ascending and descending S1 interferograms. In the top right corner are the time intervals as ddmmyy. (**C-D**) modelled S1 interferograms. The red rectangles are the sills, the red line is the dike and the blue rectangles are the faults as in Fig. S3H. The red beach-balls are the focal mechanisms from the inversion. (**E-F**) residual ascending and descending S1 interferograms. The values in the interferograms are in the satellite line-of-sight (LOS) direction and positive values mean a range increase (motion away from the satellite), such as caused by deflation. The satellite orbit and the LOS are shown by the black arrow with the tick.

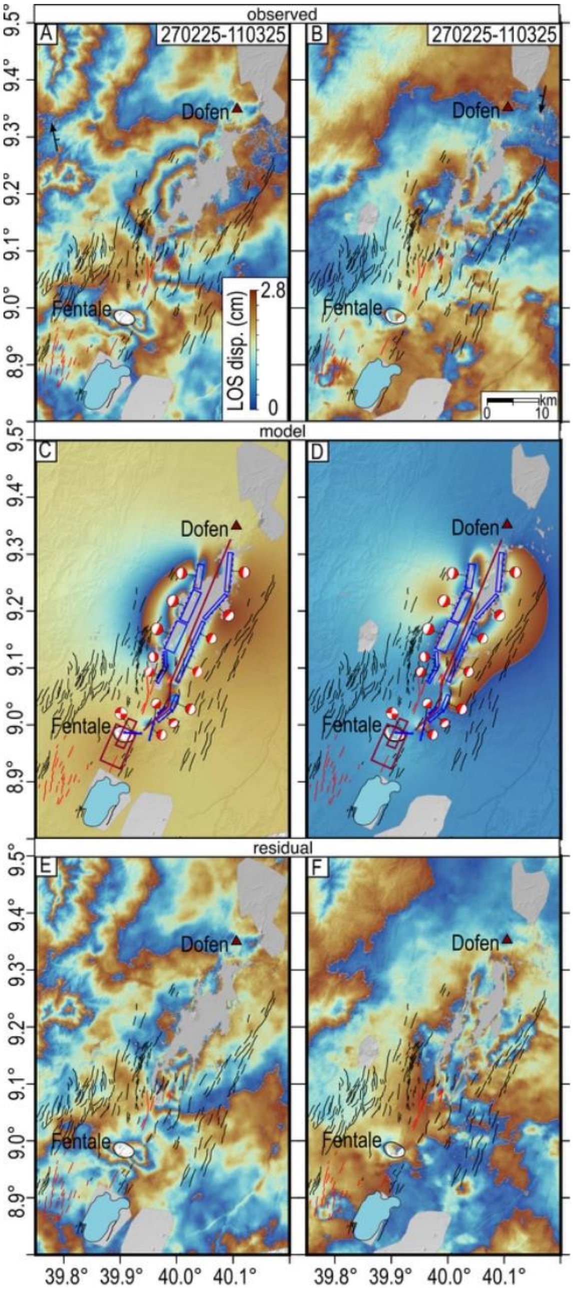

Fig. S12. InSAR modelling of 27 February 2025-11 March 2025. (**A-B**) observed ascending and descending S1 interferograms. In the top right corner are the time intervals as ddmmyy. (**C-D**) modelled S1 interferograms. The red rectangles are the sills, the red line is the dike and the blue rectangles are the faults as in Fig. S1I. The red beach-balls are the focal mechanisms from the inversion. (**E-F**) residual S1 interferograms. The values in the interferograms are in the satellite line-of-sight (LOS) direction and positive values mean a range increase (motion away from the satellite), such as caused by deflation. The satellite orbit and the LOS are shown by the black arrow with the tick.

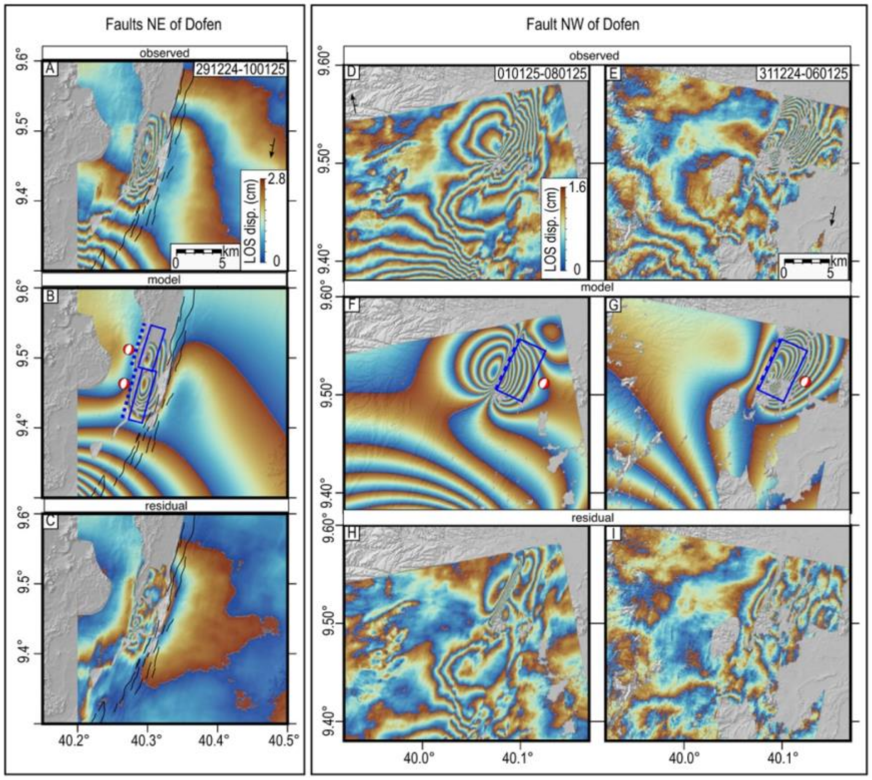

Fig. S13. InSAR modelling of faulting NE and NW of Dofen using S1 interferograms. (**A**) observed descending S1 interferogram spanning the period 29 December 2024-10 January 2025. In the top right corner is the time interval as ddmmyy. The black arrow gives the satellite orbit trajectory with the black tick marking the viewing geometry. (**B**) modelled descending S1 interferogram. The blue rectangles are the faults, as in Fig. S1C, and the dashed blue lines are the projection onto the surface of the upper edge of the faults. The red beach-balls are the focal mechanisms from the inversion. (**C**) residual descending S1 interferogram. (**D-E**) observed ascending and descending S1 interferograms spanning the period 31 December 2024-08 January 2025. In the top right corner are the time intervals as ddmmyy. The values in the interferograms are in the satellite line-of-sight (LOS) direction and positive values mean a range increase (motion away from the satellite), such as caused by deflation. The satellite orbit and the LOS are shown by the black arrow with the tick.(**F-G**) modelled ascending and descending S1 interferograms. The blue rectangle is the fault, as in Fig. S1C, and the dashed blue line is the projection onto the surface of the upper edge of the fault. The red beachball is the focal mechanism from the inversion. (**H-I**) residual ascending and descending S1 interferograms.

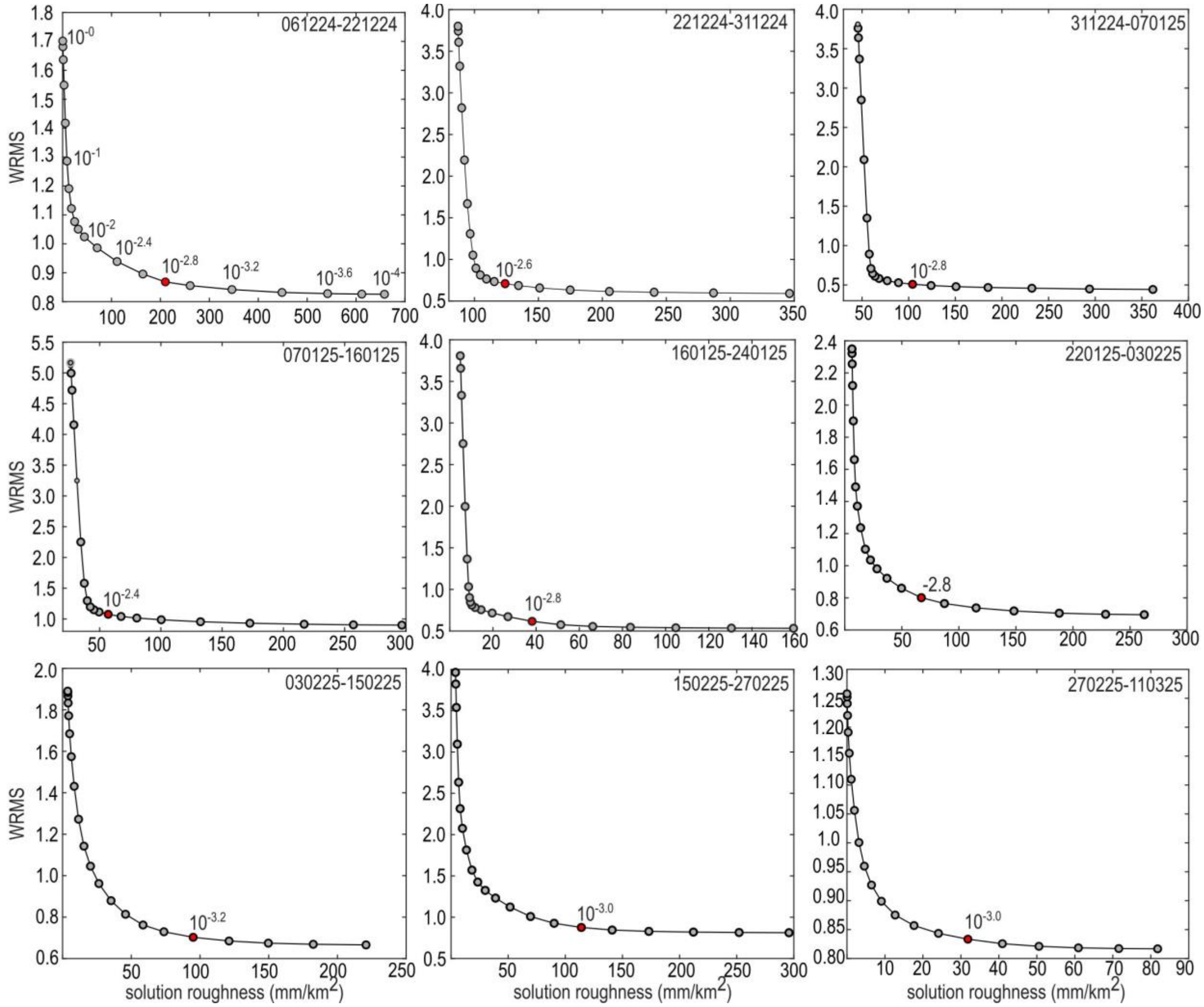

Fig. S14. Smoothing of the InSAR inversion. Trade-off curves between normalized root mean square misfit (WRMS) and solution roughness for each time interval, marked in top right corner as ddmmyy. The WRMS is the RMS weighted by the inverse variance and it is dimensionless. An WRMS ~1 indicates the model fits the data well within the estimated error variance.The red points mark the chosen smoothing factors.

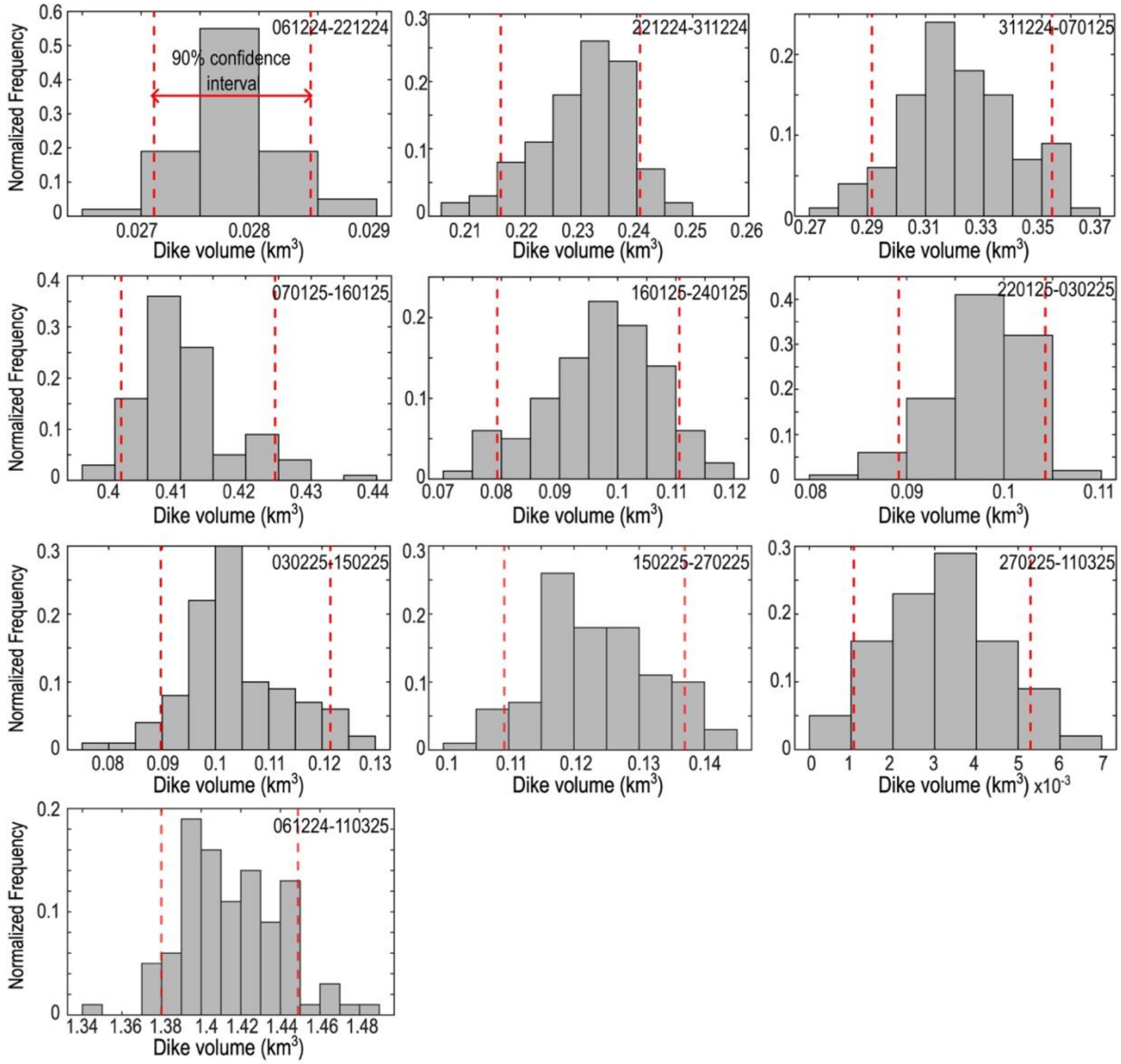

Fig. S15. Dike volume uncertainties. The 90% confidence intervals, for each time period (top three rows) and for the entire intrusion (bottom row). Histograms summarize the 100 best-fit dike volumes for the noisy data set. The 90% confidence intervals are marked as vertical dashed red lines. Each time interval is marked in top right corner as ddmmyy.

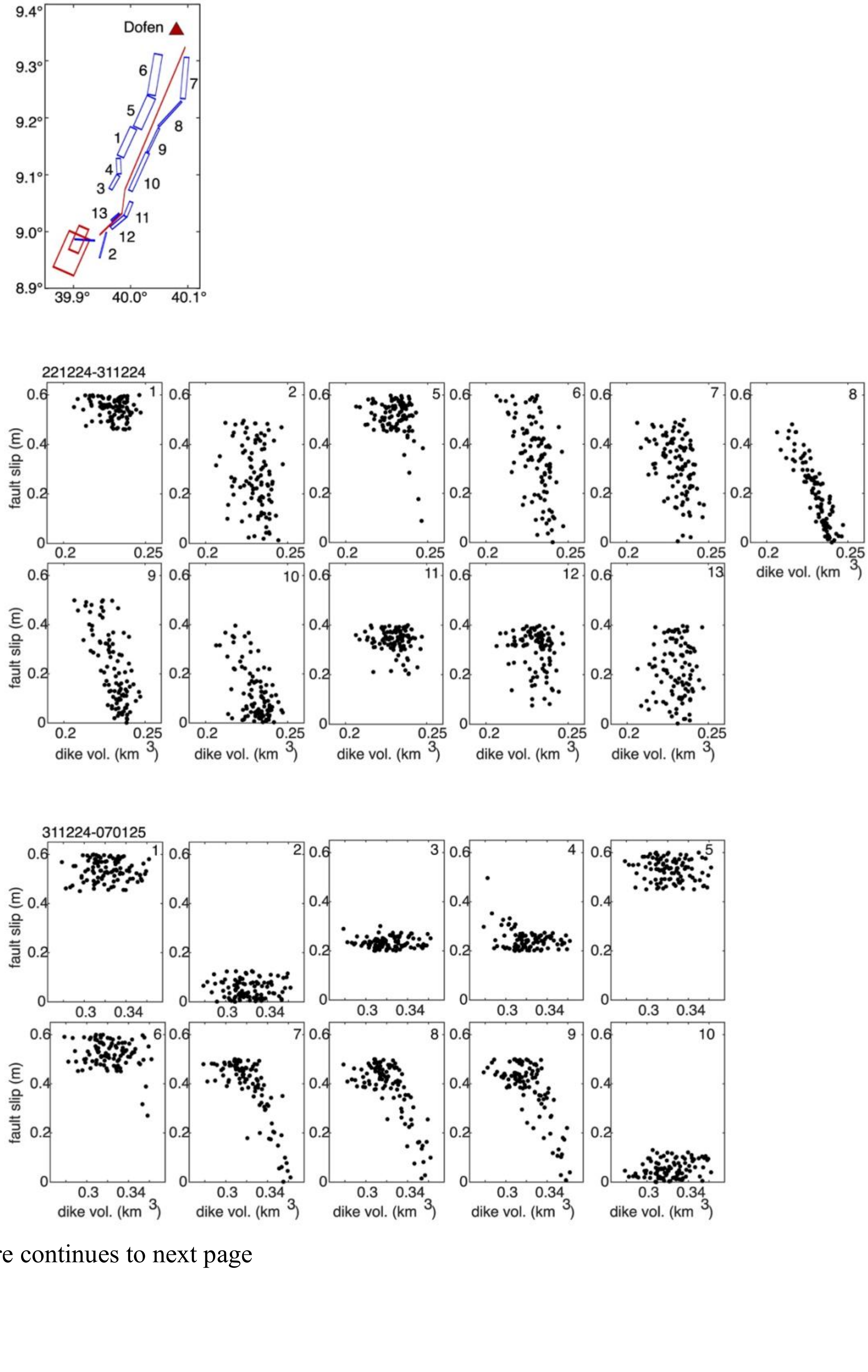

Figure continues to next page

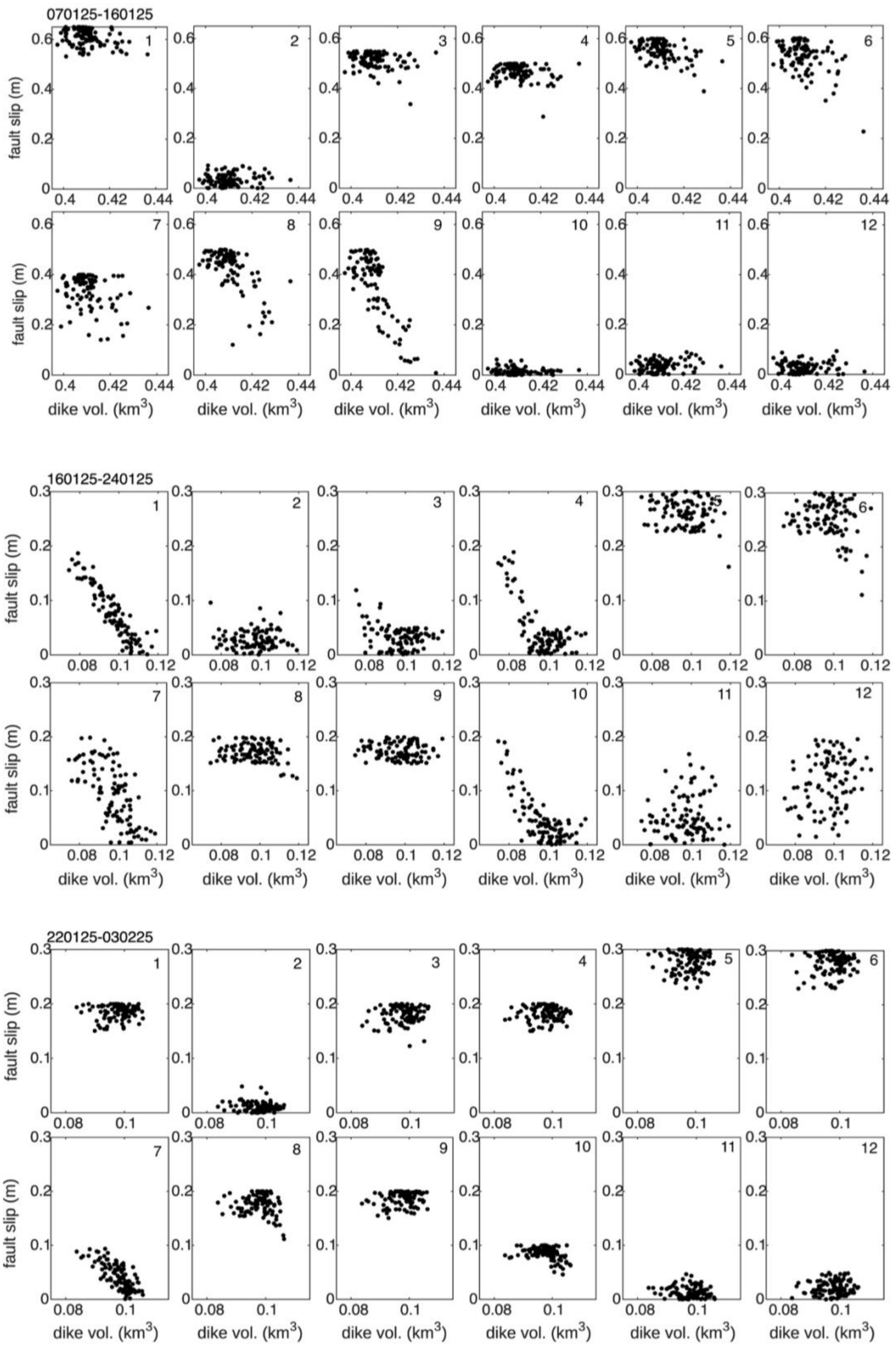

Figure continues to next page

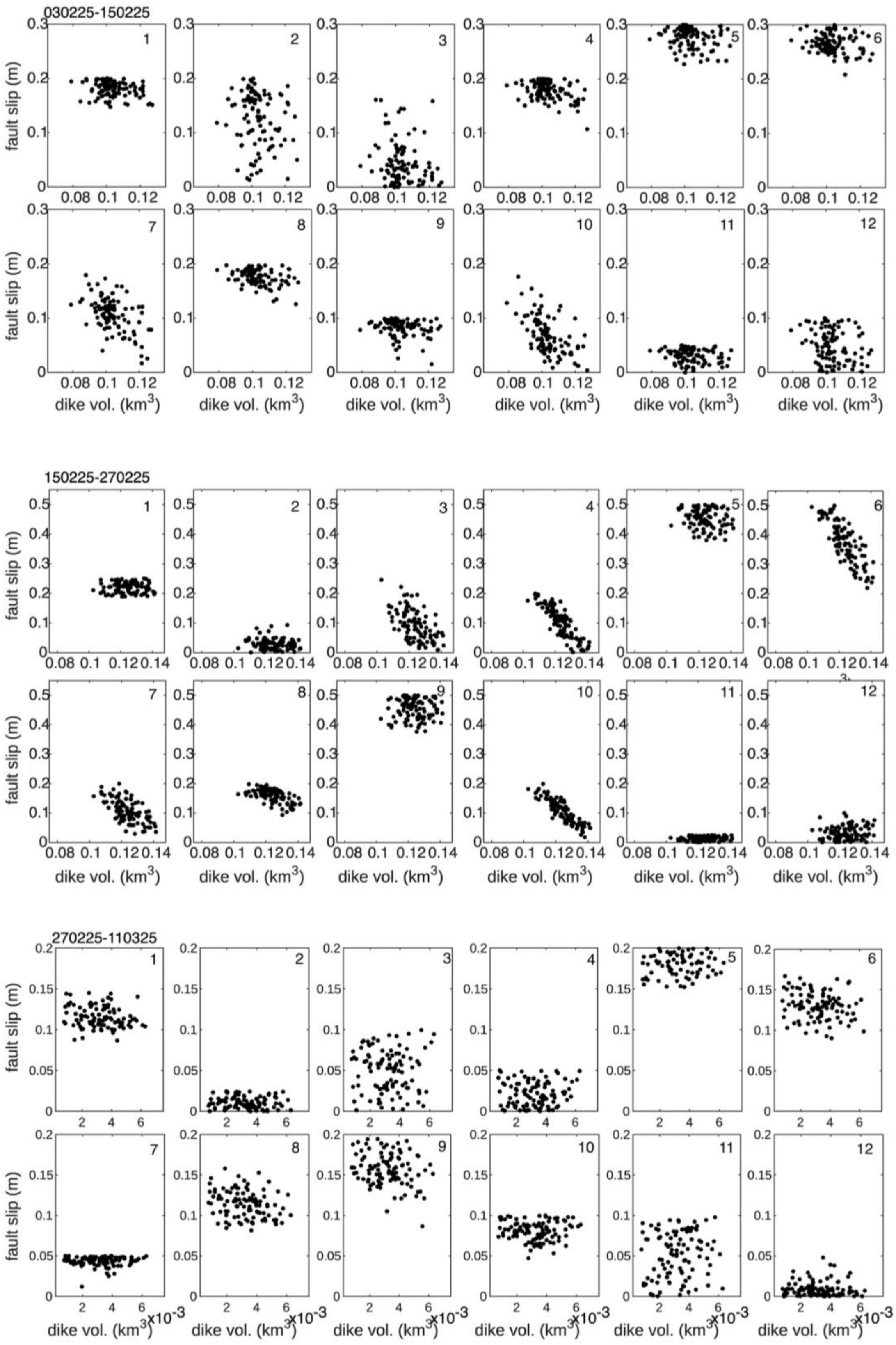

Fig. S16. Trade-off curves between dike volume and fault slip. The parametrs trade-off are shown for each time period. The location of the faults is marked in the first page of the figure.

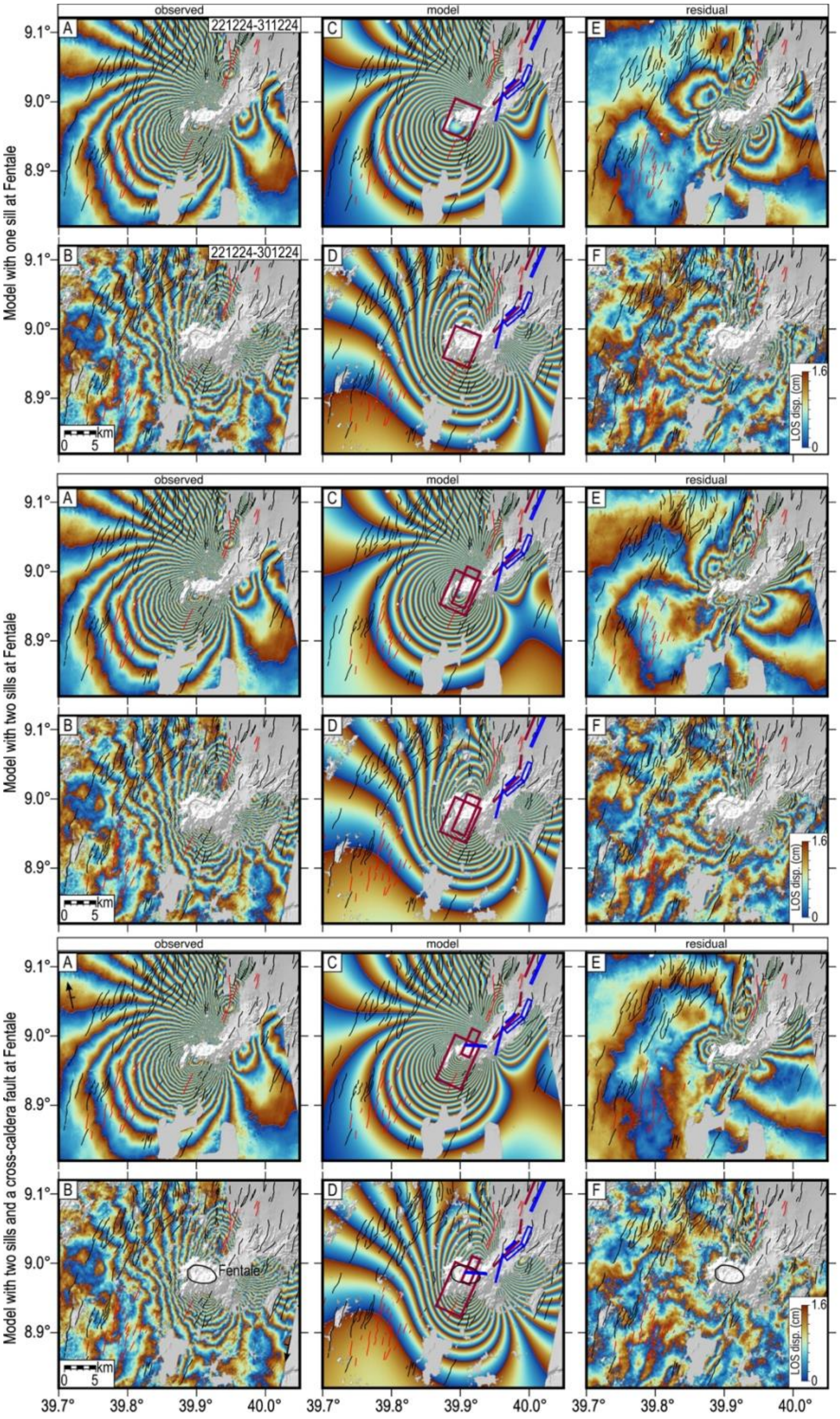

Fig. S17. Modelling of Fentale with one sill, two sills and two sills and a cross-caldera fault. In the top right corner of (A, B) are the time intervals as ddmmyy. The red rectangles are the sills, the red line is the dike and the blue rectangles are the faults. The values in the interferograms are in the satellite line-of-sight (LOS) direction and positive values mean a range increase (motion away from the satellite).

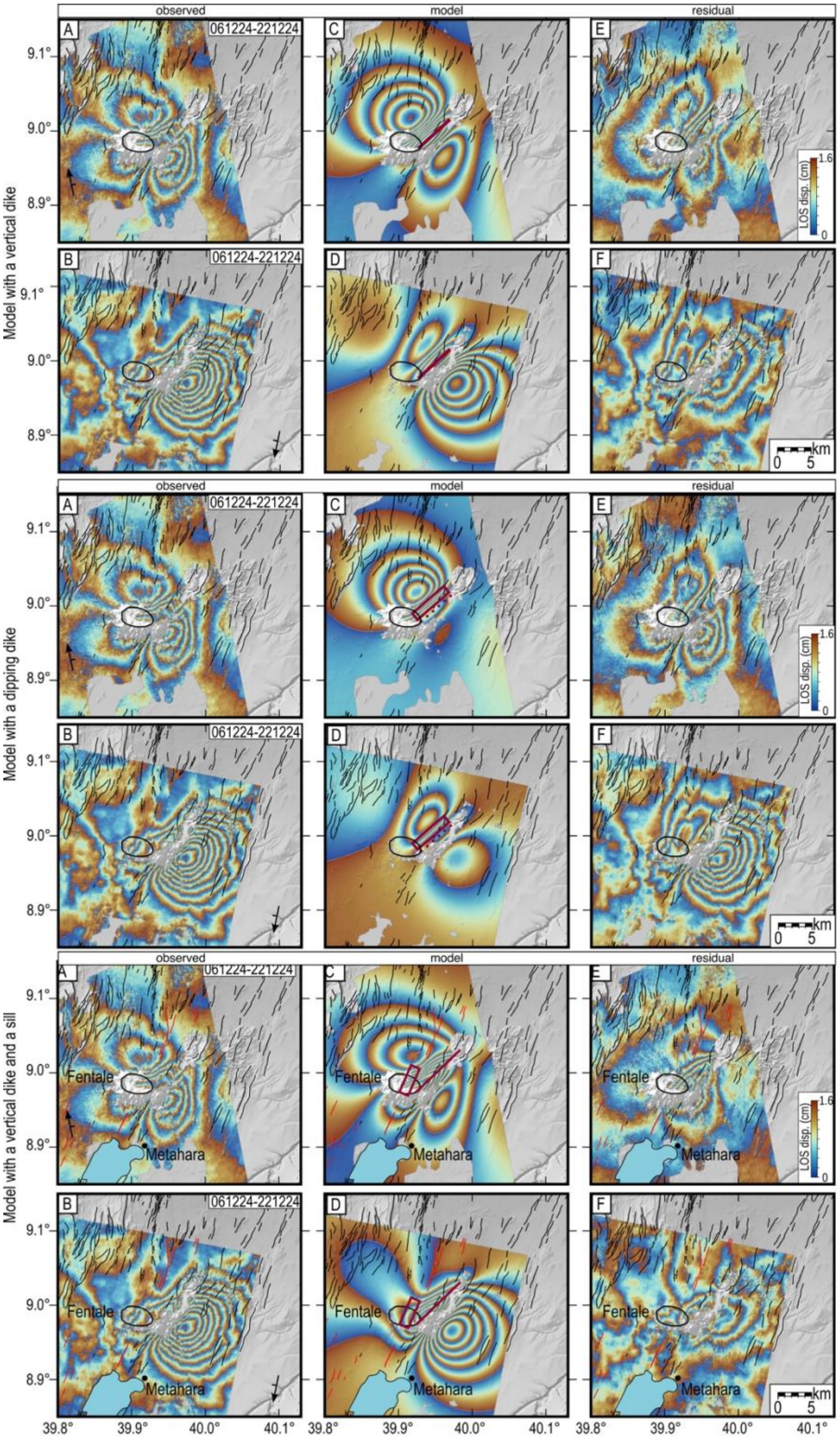

Fig. S18. Modelling of the beginning of the intrusion. In the top right corner of are the time intervals as ddmmyy. The red line in the top rows is the vertical dike, the red rectangle in the mid rows is the dipping dike with the projection at the surface of the upper edge marked by dotted line. The red rectangle in the bottom rows is the sill and the red line is the vertical dike.

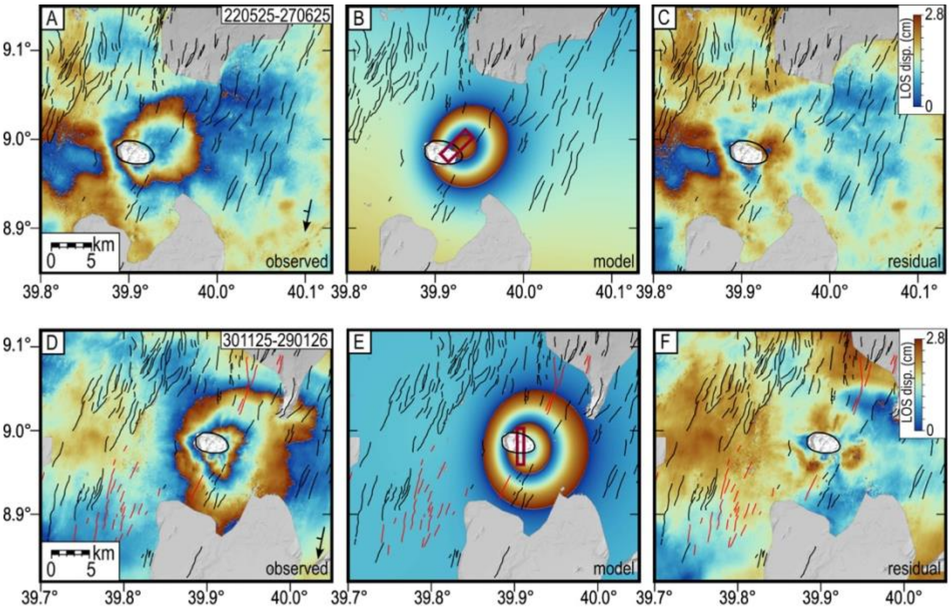

Fig. S19. Modelling of the Fentale post-intrusion inflation. In the top right corner of (**A, D**) are the two different time intervals of the descending S1 interferograms as ddmmyy. The red rectangle in (**B, E**) is the sill. The best-fit sill model in the top row is at 5.8 km depth. The best-fit sill model in the bottom row is at 6.8 km depth. The values in the interferograms are in the satellite line-of-sight (LOS) direction and positive values mean a range increase (motion away from the satellite), such as caused by deflation.

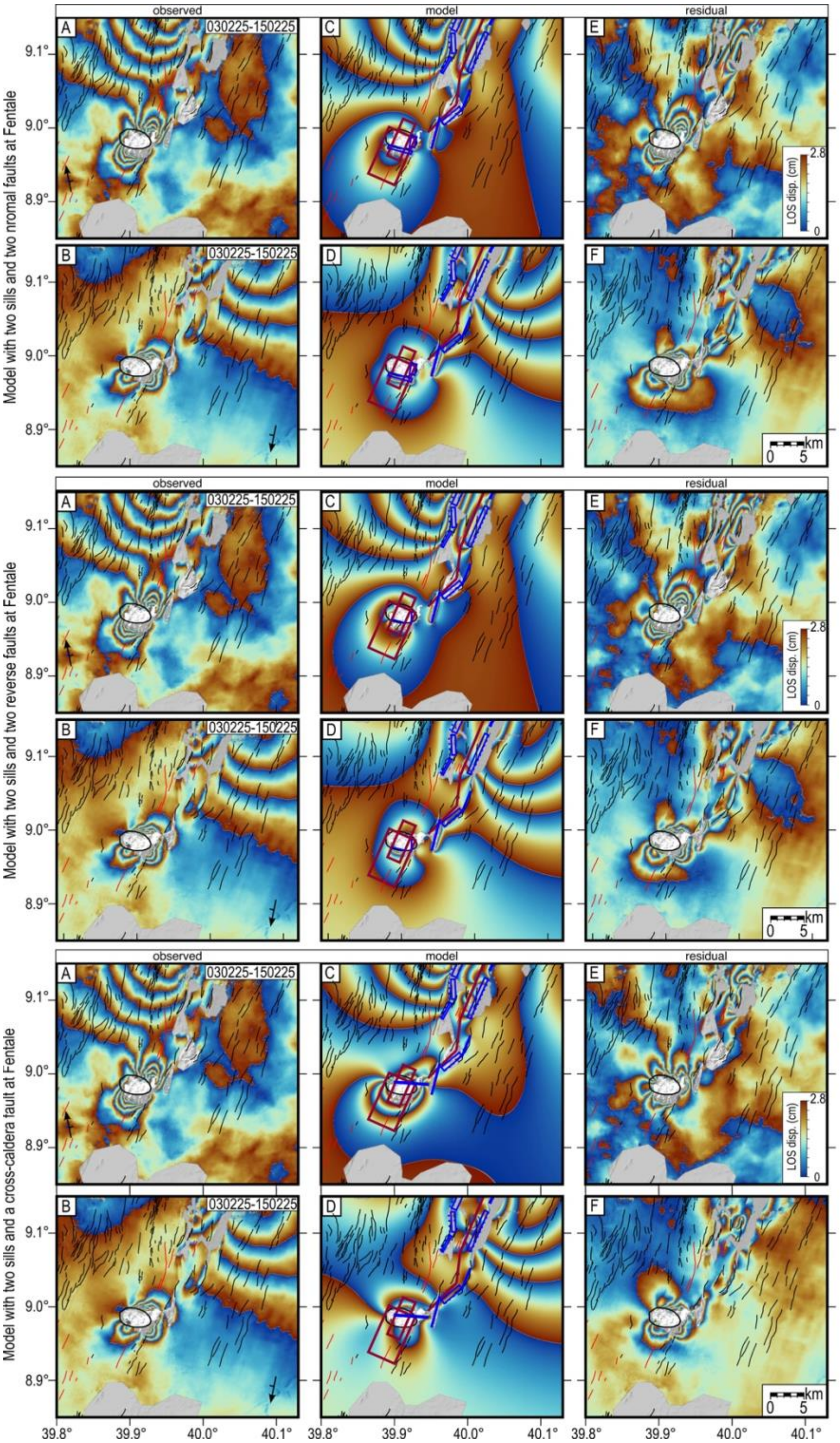

Fig. S20. Modelling of Fentale with two normal faults, two reverse faults and a cross-caldera fault. In the top right corner of (A, B) are the time intervals as ddmmyy. The red rectangles are the sills, the red line is the dike, and the blue rectangles are the faults. The values in the interferograms are in the satellite line-of-sight (LOS) direction and positive values mean a range increase (motion away from the satellite), such as caused by deflation.

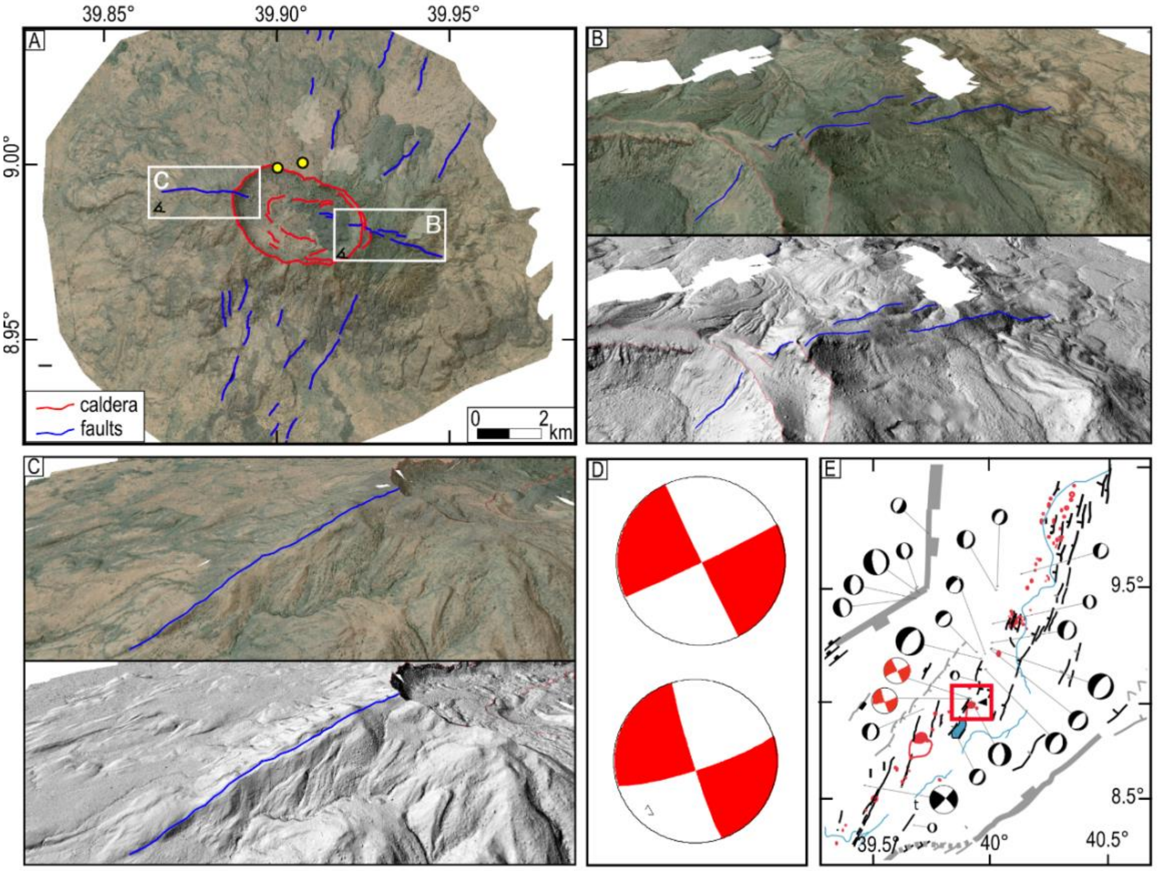

Fig. S21. Structure and earthquake focal mechanism. The data show evidence for an across rift fault that cuts through Fentale. (**A**) DEM of Fentale from Pleiades stereo panchromatic images at 50 cm resolution, showing the surface evidence of the cross-caldera fault. The yellow dots are the locations of the two strike slip focal mechanisms. (**B**) and (**C**) zoomed, oblique view of key areas where the across rift fault is best exposed. DEM from Hunt et al. (2019) (*24*). (**D**) the two strike slip focal mechanisms at Fentale that recorded from a dense temporary seismic network during 2001-2003 (*21*). (**E**) regional structural map and focal mechanisms in Keir et al. (2006) (*25*). At Fentale the focal mechanisms are strike slip and then along the Fentale-Dofen segment the focal mechanisms are normal. The position of panel (**A**) is shown with the red box.

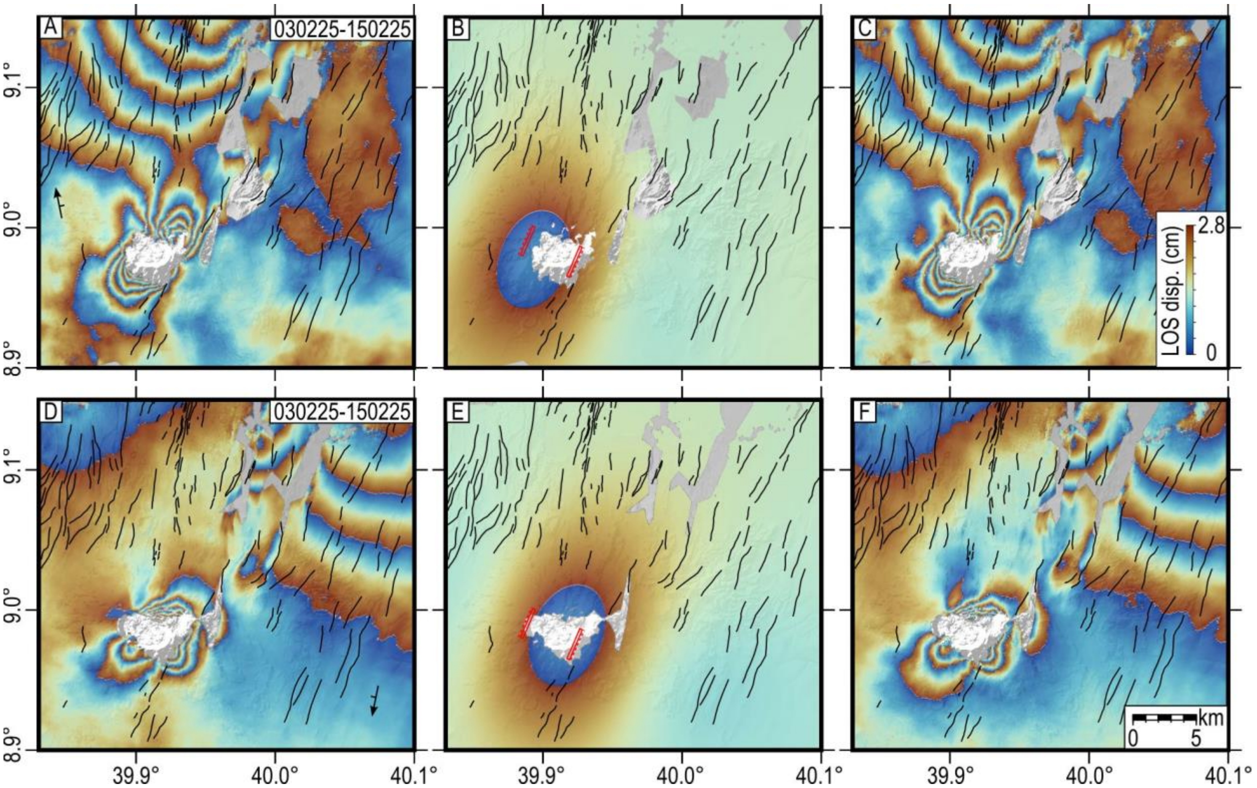

Fig. S22. Forward model assuming only conjugate normal faulting. The model is equivalent to a M_w 5.8 at depth of 12 km. (**A, D**) observed ascending and descending S1 interferograms. In the top right corner are the time intervals as ddmmyy.The values in the interferograms are in the satellite line-of-sight (LOS) direction and positive values mean a range increase (motion away from the satellite), such as caused by deflation. The satellite orbit and the LOS are shown by the black arrow with the tick. (**B, E**) ascending and descending modelled S1 interferograms assuming only conjugate normal faulting equivalent to a M_w 5.8 at 12 km depth. The red rectangles are the faults. (**C, F**) ascending and descending residual S1 interferograms showing that the deformation signal from the conjugate normal faulting at 12 km would not be detected beyond the deformation of the shallow sources.

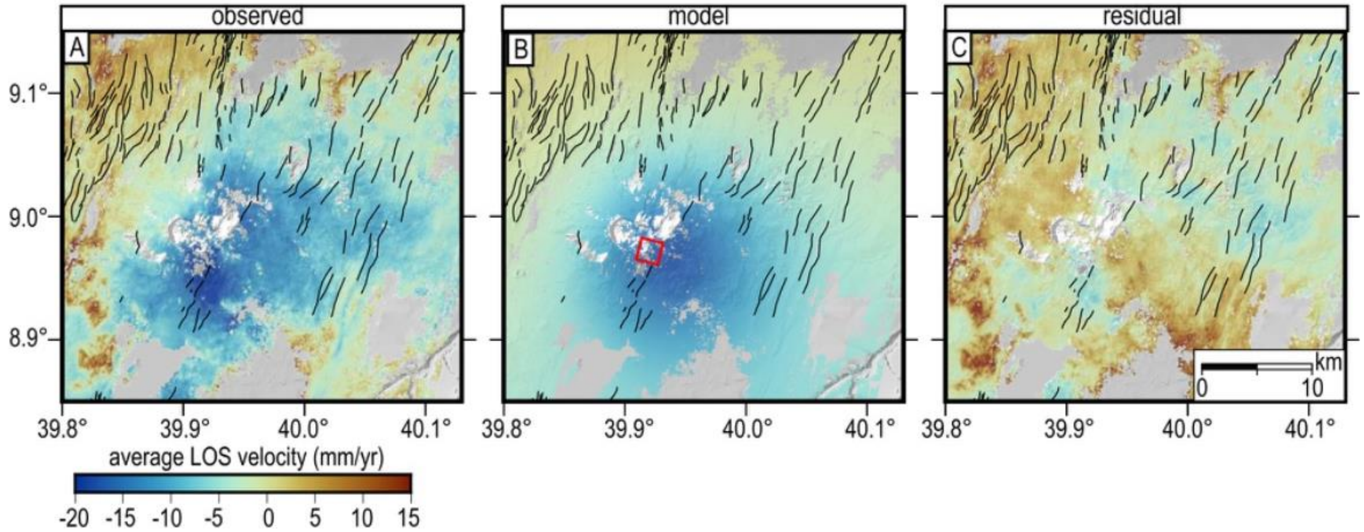

Fig. S23. InSAR modelling of the pre-diking inflation at Fentale. The figures show InSAR velocities from 7 January 2021 to 12 September 2024. (**A**) map of average InSAR velocity between 7 January 2021-12 September 2024, showing range decrease at Fentale, consistent with uplift. (**B**) InSAR model assuming an inflating sill 2 km wide by 2 km long at 12.6 km depth under Fentale (red square). (**C**) residual. The colorbar in the bottom left corner applies to all panels. The values in the interferograms are in the satellite line-of-sight (LOS) direction and positive values mean a range increase (motion away from the satellite), such as caused by deflation.

Satellite	Geometry	Track	Reference-Secondary images (ddmmyy)	Processing
S1	Descending	79	171224-291224	InSAR
S1	Descending	79	291224-100125	InSAR
S1	Descending	79	100125-220125	InSAR
S1	Descending	79	220125-030225	InSAR
S1	Descending	79	030225-150225	InSAR
S1	Descending	79	150225-270225	InSAR
S1	Descending	79	270225-110325	InSAR
S1	Ascending	87	051224-100125	InSAR
S1	Ascending	87	100125-220125	InSAR
S1	Ascending	87	220125-030225	InSAR
S1	Ascending	87	030225-150225	InSAR
S1	Ascending	87	150225-270225	InSAR
S1	Ascending	87	270225-110325	InSAR
CSK	Ascending	HI-02	061224-221224	InSAR
CSK	Ascending	HI-02	221224-311224	InSAR
CSK	Ascending	HI-02	311224-070125	InSAR
CSK	Ascending	HI-02	070125-080125	InSAR
CSK	Ascending	HI-02	070125-160125	InSAR
CSK	Ascending	HI-02	080125-160125	InSAR
CSK	Ascending	HI-02	160125-230125	InSAR
CSK	Ascending	HI-02	230125-240125	InSAR
CSK	Ascending	HI-02	240125-010225	InSAR
CSK	Ascending	HI-02	010225-080225	InSAR
CSK	Ascending	HI-02	010225-090225	InSAR
CSK	Ascending	HI-02	080225-090225	InSAR
CSK	Ascending	HI-02	090225-170225	InSAR
CSK	Ascending	HI-02	170225-240225	InSAR
CSK	Ascending	HI-02	170225-250225	InSAR
CSK	Ascending	HI-02	240225-250325	InSAR
CSK	Ascending	HI-02	250225-050325	InSAR
CSK	Descending	HI-04	061224-211224	InSAR
CSK	Descending	HI-04	061224-221224	InSAR
CSK	Descending	HI-04	211224-221224	InSAR
CSK	Descending	HI-04	221224-301224	InSAR

CSK	Descending	HI-04	301224-060125	InSAR
CSK	Descending	HI-04	060125-070125	InSAR
CSK	Descending	HI-04	060125-150125	InSAR
CSK	Descending	HI-04	070125-150125	InSAR
CSK	Descending	HI-04	150125-230125	InSAR
CSK	Descending	HI-04	230125-310125	InSAR
CSK	Descending	HI-04	310125-070225	InSAR
CSK	Descending	HI-04	310125-080225	InSAR
CSK	Descending	HI-04	070225-080225	InSAR
CSK	Descending	HI-04	070225-230225	InSAR
CSK	Descending	HI-04	080225-230225	InSAR
CSK	Descending	HI-04	230225-040325	InSAR
CSK	Descending	HI-22	211224-291224	InSAR
CSK	Descending	HI-22	291224-050125	InSAR
CSK	Ascending	HI-02	061224-221224	RO
CSK	Ascending	HI-02	221224-311224	RO
CSK	Ascending	HI-02	311224-070125	RO
CSK	Ascending	HI-02	070125-160125	RO
CSK	Descending	HI-04	061224-221224	RO
CSK	Descending	HI-04	221224-301224	RO
CSK	Descending	HI-04	301224-060125	RO
CSK	Descending	HI-04	070125-150125	RO
CSK	Descending	HI-22	211224-291224	RO
**CSK**	**Descending**	**HI-22**	**291224-050125**	**RO**

Table S1. List of SAR data used in this study. Both InSAR and maps of RO (range offsets) were processed. A selection of the InSAR and RO were used for the kinematic modelling (Fig. S3-S13).
